# Direct airway delivery of a humanized anti-H7N9 neutralizing antibody broadly protects against divergent H7 influenza viruses in the mouse model

**DOI:** 10.1128/jvi.01327-25

**Published:** 2025-11-24

**Authors:** Wang Yu, Xiaozheng He, Jiangyan Zhao, Yunlong Dou, Tingyu Hu, Xia Chen, Xuran Ma, Xiaoquan Wang, Shunlin Hu, Jiao Hu, Xiufan Liu, Zenglei Hu

**Affiliations:** 1 Key Laboratory of Avian Bioproducts Development, Ministry of Agriculture and Rural Affairs, Yangzhou University, College of Veterinary Medicine 38043, Yangzhou, China; 2 Joint International Research Laboratory of Agriculture and Agri-Product Safety, The Ministry of Education of China, Yangzhou University 38043, Yangzhou, China; Emory University School of Medicine, Atlanta, Georgia, USA

**Keywords:** H7 subtype influenza virus, neutralizing monoclonal antibody, airway delivery, humanized antibody, broad protection

## Abstract

Passive administration of broadly neutralizing anti-influenza monoclonal antibodies (mAbs) before or after virus infection can prevent or alleviate disease. Unlike seasonal influenza, infection with zoonotic avian influenza viruses can lead to acute respiratory distress syndrome and high mortality in humans. Respiratory tract-targeting antibody delivery appears to be more clinically relevant and effective for zoonotic influenza treatment. In this study, the efficacy of an anti-H7N9 murine mAb 4B7 and its humanized form (chi4B7) against H7 subtype influenza viruses administered through the intranasal route was investigated in mice. 4B7 recognizes critical residues in the vestigial esterase domain and receptor-binding sites in the hemagglutinin of H7N9 virus. The antibody had cross-H7 binding, hemagglutination inhibition, and neutralizing activities. In particular, the dose of 4B7 required for prophylactic protection against H7N9 infection was significantly reduced in mice treated locally (intranasal) compared with those treated systemically (intraperitoneal). Intranasal delivery of the antibody also enhanced therapeutic efficacy against H7N9 infection compared to intraperitoneal administration. Chi4B7 generated by grafting the variable regions onto the human IgG1 backbone sustained cross-reactivity with different H7 viruses of the parental murine antibody. Airway-delivered chi4B7 provided broad prophylactic and therapeutic protection against divergent H7 viruses in mice. Moreover, intranasal administration of chi4B7 had a long effective prophylaxis window against H7N9 infection. Our results suggest that airway delivery of the humanized anti-H7 antibody is a favorable approach for broad-spectrum prophylaxis and therapy against the H7 subtype influenza.

**IMPORTANCE:**

Infection of zoonotic H7 avian influenza viruses can cause severe respiratory symptoms and high mortality in humans. Monoclonal antibody administration is an effective approach for treatment of zoonotic influenza infection, while systematic routes of antibody administration (typically intravenous infusion) have several shortcomings. However, there are no approved anti-H7 antibody therapies, and the efficacy of antibodies administered through the airway route against H7 viruses has not been fully investigated. Herein, we report a murine broadly neutralizing monoclonal antibody against divergent H7 viruses and reveal that intranasal administration enhanced prophylactic and therapeutic efficacy of this antibody against H7N9 virus compared to systemic administration. Airway delivery of the humanized antibody conferred broad protection against diverse strains of H7 virus in mice. Our study presents new candidates of broad antiviral agents against H7 avian influenza viruses and highlights airway delivery as a more potent manner of administering antibodies for clinical treatment of influenza.

## INTRODUCTION

Avian influenza viruses (AIVs) pose a significant threat to both animal and human health due to their ability to cross species barriers and cause zoonotic infections (1). Among AIVs, the highly pathogenic H5N1 subtype (clade 2.3.4.4b) has recently caused an unprecedented outbreak in dairy cows and other mammalian animals in the United States (2, 3), with spillover events leading to dozens of confirmed human infections (4). While H5N1 has garnered significant attention, the H7 subtype AIVs also circulate and cause disease outbreaks in poultry worldwide (5–9). In addition, the H7 subtype AIVs have also emerged as a public health concern. Between 2013 and 2017, five epidemic waves of H7N9 virus in China resulted in over 1,500 laboratory-confirmed human infections, resulting in severe respiratory illness and a mortality rate approaching 40% (1). In addition to the H7N9 subtype, viruses of the H7N2, H7N3, H7N4, and H7N7 subtypes also cause human infection cases with mild-to-severe respiratory symptoms (1, 7). In 2018, a human case infected with a novel H7N4 subtype AIV was reported in China (10). Currently, H7N4 viruses are circulating in shorebirds, and a strain is pathogenic in mice without prior adaptation, indicating a potential risk to other mammals and humans (11). Humans remain immunologically naïve to the H7 subtype AIVs, raising concerns that zoonotic H7 viruses could acquire pandemic potential through adaptation to human hosts. Therefore, it is vital to develop effective antiviral agents against the H7 subtype viruses.

Despite ongoing efforts to develop vaccines against the H7 subtype AIVs (12–14), no licensed vaccines are currently available for human use. Moreover, existing vaccination strategies of H7 vaccines often induce suboptimal levels of hemagglutination inhibition (HI) and virus-neutralizing (VN) antibodies (15, 16), underscoring the need for improved vaccine design and optimization. Moreover, the efficacy of vaccines is impaired in immunocompromised and elderly populations. For severe influenza infections, including those caused by zoonotic H5 and H7 viruses, effective antiviral therapeutics are critical for reducing morbidity and mortality (17, 18). While the neuraminidase inhibitors (such as oseltamivir) and the cap-dependent endonuclease inhibitor (baloxavir marboxil) are currently used to treat influenza, their efficacy is limited by time-sensitive administration and the emergence of drug-resistant virus strains (17, 19). Administration of neutralizing monoclonal antibodies (mAbs) is an attractive alternative for influenza treatment, especially for immunocompromised and elderly populations and individuals who are at high risk of virus exposure (20, 21). Moreover, mAbs targeting the conserved neutralizing epitopes are desired to protect against diverse influenza viruses.

MAbs have emerged as a promising approach for prophylaxis and therapy of viral infections, with several mAb therapies already approved for severe acute respiratory syndrome coronavirus 2 (SARS-CoV-2) and Ebola virus infections (22). Notably, numerous antibodies against the H7 subtype AIVs have demonstrated potent antiviral activity in preclinical studies (23–26). Perhaps due to the effectiveness of systematic passive immunization using infused antisera, the general approach for delivering mAbs has been through the systemic route, mainly intravenous infusion. However, influenza virus infection is initiated in and mainly restricted to the respiratory tract, and thus local administration of anti-influenza mAbs to the patient’s airway appears to be a clinically relevant and effective approach. Moreover, the concentration of systematically administered antibodies in the respiratory tract is low, and thus a high concentration of antibodies are needed for protection from lethal infection. Both the manufacturing process and the antibody amount required for protection make mAb therapy expensive and inaccessible for large-scale implementation. Previous studies have shown that direct administration in the respiratory tract can improve the efficacy of broadly neutralizing mAbs against influenza A (H1N1, H1N1pdm, H3N2, and H5N1) and B viruses compared to the systematic route of administration (27, 28). Infection of zoonotic H5 or H7 viruses can lead to acute respiratory distress syndrome (ARDS) and high mortality, while the efficacy of anti-H7 mAbs through airway delivery remains to be fully assessed.

Antibody humanization and human antibody production are important approaches in passive immune therapy because murine antibodies can trigger a human anti-mouse antibody response (29). Therefore, minimizing the murine immunogenic components in antibodies while preserving the functional antigen-binding domains of the original murine antibodies is a common strategy for the design of therapeutic antibodies. Several human antibodies against the H7N9 subtype AIV were generated from vaccinated-healthy donors or virus-infected patients (23–25, 30). However, the source of human specimens is limited, and collection and handling of such samples are strictly regulated. Therefore, humanization of murine antibodies is still an indispensable method for developing clinically relevant antibodies.

Although there are many reports on the generation of antibodies against H7 viruses, studies on airway delivery and humanization of anti-H7 mAbs are limited. In this study, a neutralizing murine mAb 4B7 against the hemagglutinin (HA) of the H7N9 subtype AIV was generated. This antibody targets the vestigial esterase domain (VED) and receptor-binding sites (RBS) in H7 HA and can cross-react to divergent H7 viruses. Systemic and intranasal administration of the antibody conferred protection against H7N9 virus infection in mice, while airway delivery significantly enhanced the efficacy of the antibody. A humanized antibody generated by grafting the variable regions onto a human IgG1 backbone sustained antigen binding, HI, and neutralizing activities of the murine antibody. More importantly, airway delivery of the humanized antibody provided broad protection against H7N9, H7N4, and H7N3 viruses. Our results suggest that the airway delivery of a humanized anti-H7 neutralizing antibody could be an effective approach to protect against divergent H7 subtype influenza viruses.

## RESULTS

### Generation and characterization of H7N9-reactive mAbs

**Fig 1:**
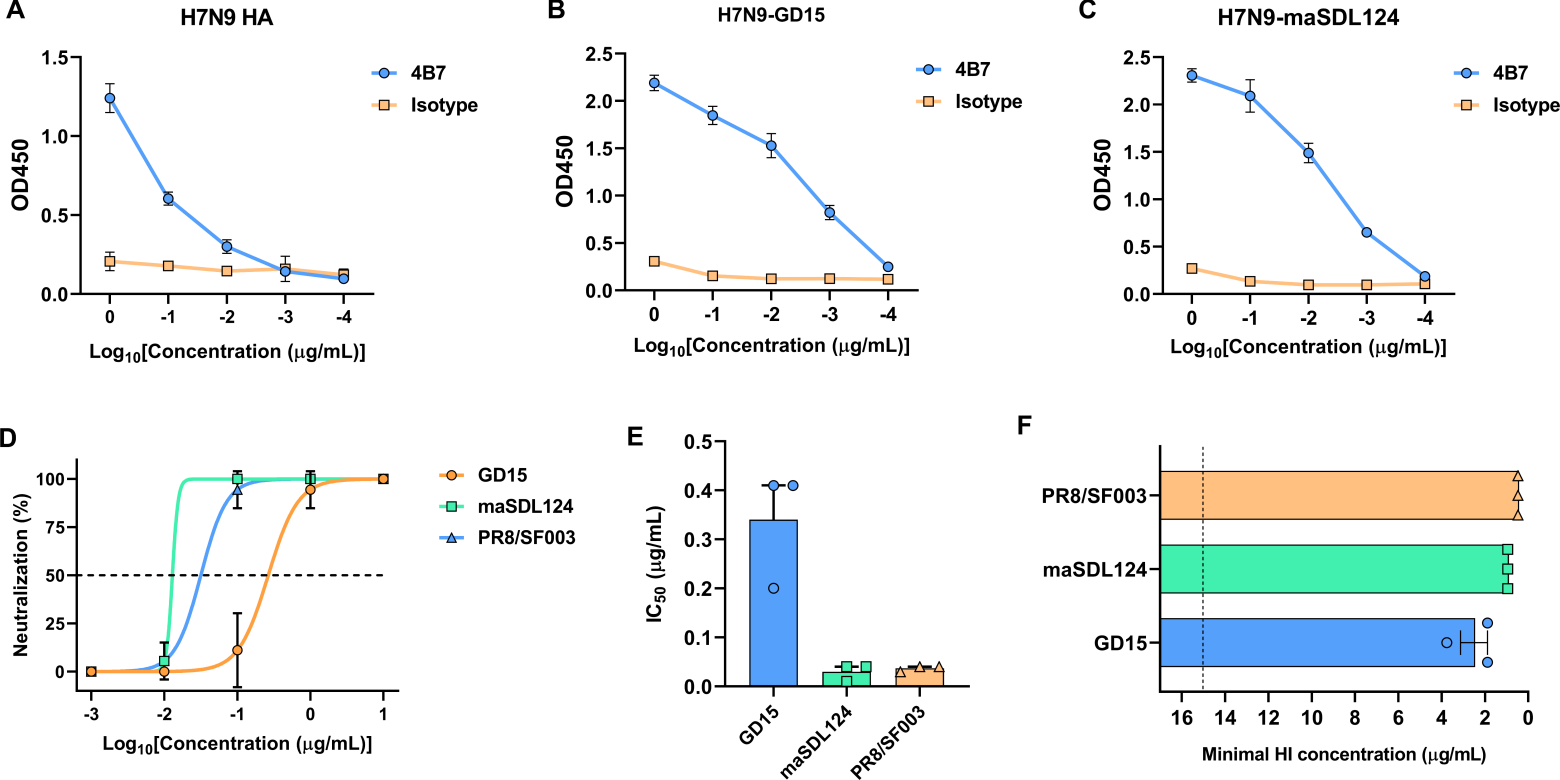
Binding, neutralization, and hemagglutination-inhibition activities of the mAb 4B7 with H7N9 virus. (**A–C**) Binding activities of the antibody to the H7N9 HA protein (**A**), H7N9 GD15 strain (**B**), and maSDL124 strain (**C**). (**D**) Neutralization rates of the antibody against three H7N9 viruses. (**E**) The half-maximal inhibitory concentration (IC_50_) values of the antibody against H7N9 viruses. The dashed line indicates 50% neutralization. (**F**) Hemagglutination inhibition (HI) activities of the antibody against H7N9 viruses. The dashed line indicates the starting antibody concentration (15 µg/mL). Mean values ± standard error of the mean (SEM) of three independent experiments are shown.

To screen neutralizing mAbs against H7N9 virus, the frozen hybridomas prepared in our previous study (31) were recovered and screened using enzyme-linked immunosorbent assay (ELISA) and microneutralization test. After a three-round screening, a mAb 4B7 with potent neutralizing activity against H7N9 virus was obtained. Minimal binding concentrations of the mAb with the H7N9 HA protein and H7N9 viruses were 0.01 and 0.001 µg/mL, respectively, indicating high binding affinity of the antibody to the antigens (Fig. 1A through C). VN and HI titers of the antibody against three H7N9 viruses were determined, and amino acid identity of the HA protein among these viruses is above 97% (Fig. S1A). Half-maximal inhibitory concentrations (IC_50_) of 4B7 against a mouse-adapted A/chicken/China/SDL124/2015 (maSDL124), PR8/SF003 (a PR8 6:2 reassortant with the HA and NA genes deriving from A/Guangdong/17SF003/2016), and A/chicken/Guangdong/GD15/2016 (GD15) were 0.03, 0.036, and 0.34 µg/mL, respectively (Fig. 1D and E). In addition, the minimal HI concentrations (MHC) of 4B7 against maSDL124, PR8/SF003, and GD15 were 0.94, 0.47, and 2.5 µg/mL, respectively (Fig. 1F). These results indicate that 4B7 is an H7N9 HA-reactive mAb with potent neutralizing and HI activities against H7N9 viruses.

### Interaction between 4B7 and the H7N9 HA analyzed by molecular docking

**Fig 2:**
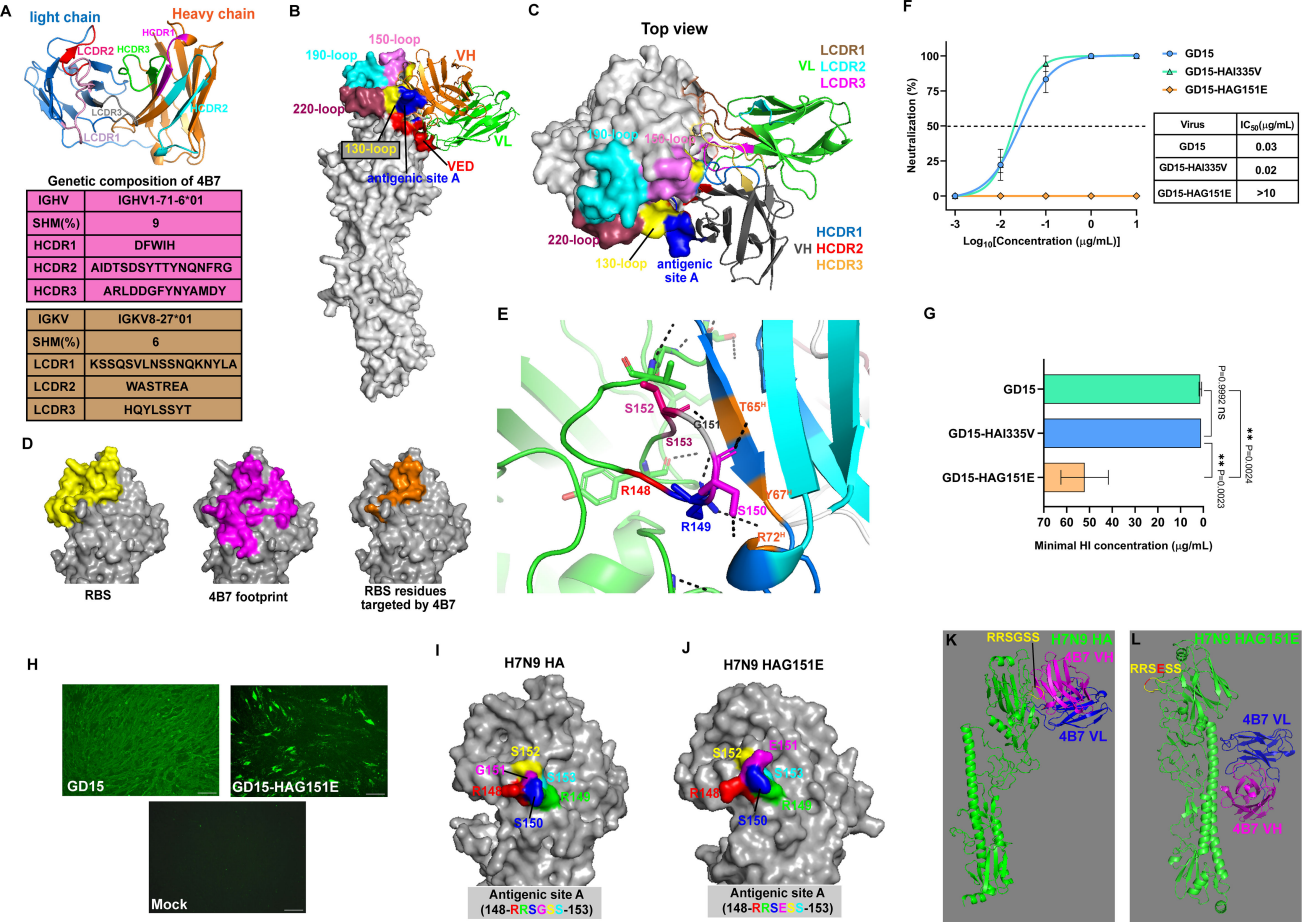
Interaction between the antibody and H7N9 HA and the escape mutant study. (**A**) Genetic signatures of the mAb 4B7. The genes encoding the variable regions of 4B7 were sequenced and the complementarity-determining regions (CDRs) in the heavy and light chains are illustrated. (**B**) Predicted docking structure between 4B7 and the H7N9 HA protein. Molecular docking of the 4B7 single-chain antibody (scFv) and the HA protein was performed using the ZDOCK server. The vestigial esterase domain (VED) and critical domains in the receptor-binding site (RBS) in the HA protein, including the 130-, 150, 190-, and 220-loops, as well as the antigenic site A, are highlighted. (**C**) Interaction between the CDRs with the HA protein. (**D**) Overlap of the RBS in HA with 4B7 footprint. The 130-, 150-, 190-, and 220-loops in the RBS are illustrated in yellow, and the 4B7 footprint is highlighted in magenta. Overlap between the RBS and 4B7 footprint is shown in orange. (**E**) Hydrogen bonds formed between the antigenic site A and the 4B7 heavy chain. The antigenic site A (148-RRSGSS-153) in HA and residues in the heavy chain (T65, Y67, and R72) are highlighted. Hydrogen bonds are shown as the black-dashed lines. (**F**) Neutralizing activity of 4B7 with the H7N9 virus and escape mutants. The dashed line stands for 50% neutralization. (**G**) Hemagglutination inhibition (HI) activity of 4B7 with the H7N9 virus and escape mutants. Two reassortant H7N9 viruses harboring I335V and G51E mutations in HA were rescued. Neutralizing and HI activities of the antibody with H7N9 viruses were measured. (**H**) Reactivity of 4B7 with H7N9 viruses determined using immunofluorescence assay. MDCK cells were inoculated with H7N9 GD15 or escape mutant GD15-HAG151E or left non-inoculated. The mAb 4B7 was used as the first antibody in immunofluorescent assay. Scale bar, 100 µm. (**I**) Conformation of the antigenic site A (148-RRSGSS-153) in HA. (**J**) Conformation of the antigenic site A with the G151E mutation (148-RRSESS-153) in HA. Molecular docking of 4B7 scFv with H7N9 HA (**K**) and HA with the G151E mutation (**L**). Mean values ± standard error of the mean (SEM) of three independent experiments are shown. The data were analyzed using one-way ANOVA with Tukey’s multiple comparison test. Asterisks stand for significant differences, and *P* values are shown. ns, nonsignificant.

Sequence analysis and isotype determination revealed that the mAb 4B7 is a murine IgG1 antibody. The antibody uses IGHV1-71-6*01 for the heavy chain with 9% somatic hypermutation (SHM) and IGKV8-27*01 for the light chain with 6% SHM (Fig. 2A). Three complementary determining regions (CDRs) in the heavy and light chains of 4B7 were identified, and CDR3 in the heavy (HCDR3) and light chains (LCDR3) are 14 and 8 amino acids, respectively (Fig. 2A). Molecular docking demonstrated that a total of 48 amino acids were present in the interface between the 4B7 single-chain antibody (scFv) and H7N9 HA, and their interactions involved the VED, RBS (130-loop and 150-loop), and the antigenic site A (Fig. 2B through D; Table S1). Most interactions are driven by the heavy chains and consist of hydrogen and salt bridge bonds. Amino acids in the HCDR1 (D36 and W38) and HCDR2 (D57, S59, D62, and S63) form hydrogen or salt bridge bonds with key residues in the 130-loop (R139, T140, N141, and V143) (Table S2). Three residues in the HCDR2 (T65, Y67, and R72) mainly bind critical residues in the 140-loop in the antigenic site A (R149, S150, and S152) through hydrogen bonds (Fig. 2E; Table S2). Y112 in the HCDR3 and S59 in the HCDR2 bind to L159 and T165 in the 150-loop via hydrogen bonds, respectively (Table S2). The light chain is less involved in interaction, and residues in the LCDR1 and LCDR3 form hydrogen bonds with the 128-KEPMG-132 motif close to the 130-loop (Table S2). These results suggest that the mAb 4B7 interacts with the VED and critical domains in the RBS of the H7N9 HA protein.

### G151E mutation in the antigenic site A mediates H7N9 virus escape from 4B7 neutralization

To validate epitope prediction by molecular docking, escape mutants were generated in chicken embryos by incubating a mixture of the H7N9 GD15 strain and 4B7 at a neutralizing concentration. After a three-round screening, there was a 16-fold reduction in the HI titer of the antibody against the escape mutants compared to that against the parental virus (Table S3). The escape mutants were plaque-purified, and two mutations, G151E and I335V, in the HA protein were identified (Table S3).

To determine the role of these mutations in virus escape, two reassortant H7N9 viruses carrying the G151E or I335V mutation in HA were generated. The mAb 4B7 had strong neutralizing activities against GD15 (IC_50_: 0.03 µg/mL) and GD15-HAI335V (IC_50_: 0.02 µg/mL) but did not neutralize GD15-HAG151E at up to 10 µg/mL (Fig. 2F). Similarly, MHCs of 4B7 against GD15 (1.31 µg/mL) and GD15-HAI335V (0.98 µg/mL) were comparable, while the MHC against GD15-HAG151E was significantly increased by around 40-fold (52.1 µg/mL) (Fig. 2G). In addition, the reactivity of the mAb 4B7 with GD15-HAG151E was markedly decreased compared to that with GD15, as determined by immunofluorescence assay (IFA) (Fig. 2H). G151 is a critical residue in the antigenic site A (148-RRSGSS-153, based on H7 numbering) in H7 HA, and mutation at this position resulted in prominent conformational changes of residues in this domain, including R148, S150, and G151 (Fig. 2I and J). Moreover, molecular docking revealed no interface between the HA protein carrying the G151E mutation and the 4B7 scFv (Fig. 2K and L), which correlated to the IFA data and validated the role of this mutation in virus escape. Therefore, the G151E mutation in the antigenic site A, a major epitope targeted by 4B7, was associated with H7N9 virus escape from 4B7 neutralization.

### The mAb 4B7 cross-reacts with diverse H7 viruses

**Fig 3:**
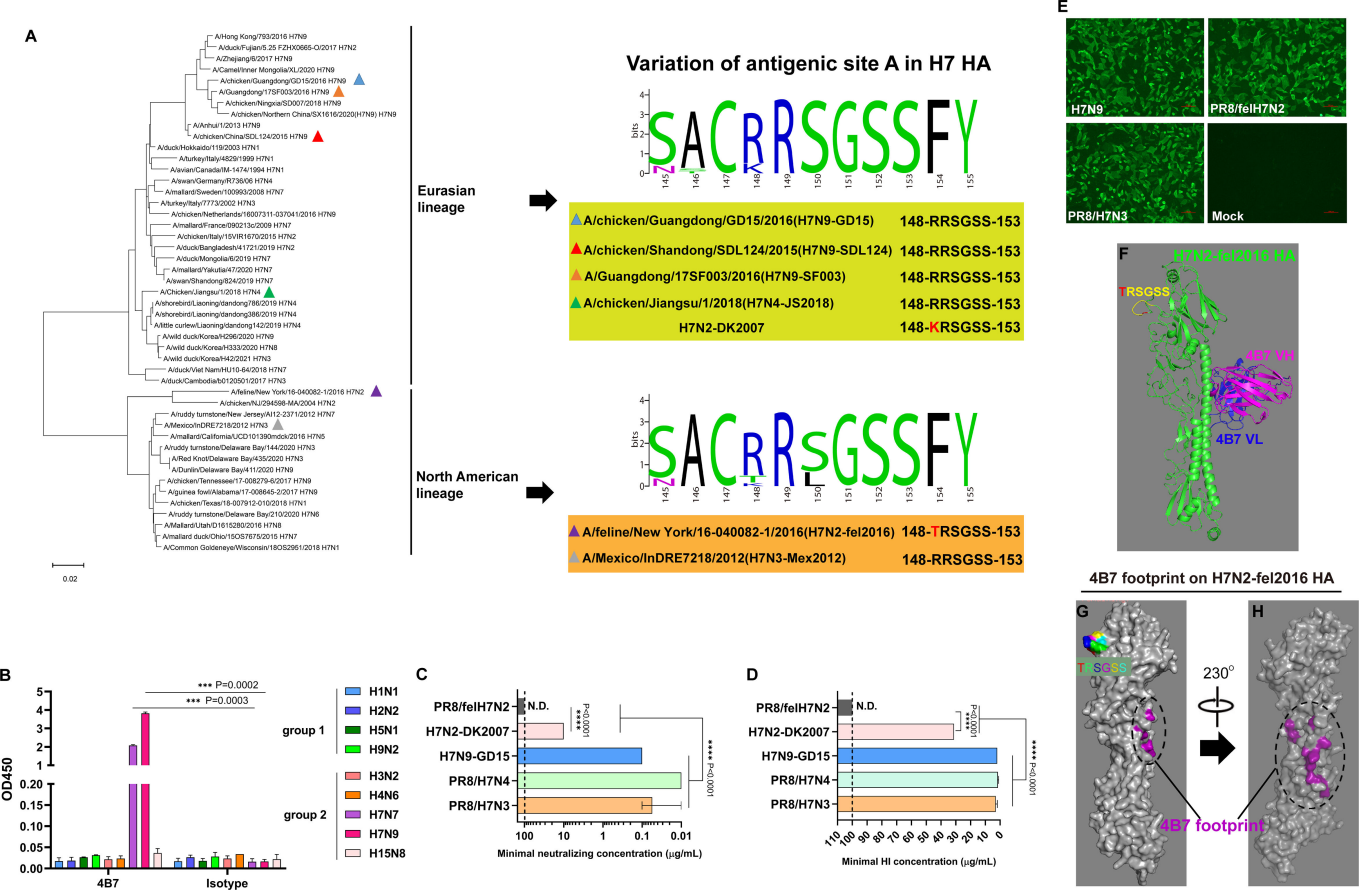
Conservation analysis of the antigenic site A and cross-reactivity of the mAb 4B7 with different H7 viruses. (**A**) Phylogenetic tree of the H7 subtype influenza viruses and conservation of the antigenic site A. The HA genes of representative H7 viruses belonging to Eurasian and North America lineages were used to generate a phylogenetic tree using the neighbor-joining method in MEGA11 software. The viruses used in this study are indicated with triangles, and variation of the antigenic site A of these strains is shown. (**B**) Cross-binding activity of the antibody with the HAs of different subtypes. Reactivity of the antibody with the HA proteins of representative subtypes in group 1 and group 2 was measured using ELISA. Mean values ± standard error of the mean (SEM) of two independent experiments are shown. (**C**) Cross-neutralizing activity of the antibody with different H7 viruses. Minimal antibody concentrations required to completely suppress virus replication in microneutralization assay are shown. (**D**) Cross-HI activity of the antibody with different H7 viruses. Minimal antibody concentrations required to completely inhibit hemagglutination of the viruses are shown. The dashed lines indicate the starting antibody concentration (100 µg/mL). For microneutralization and HI tests, mean values ± standard error of the mean (SEM) of three independent experiments are shown. N.D., not detected. In (**B through D**), asterisks indicate significant differences, and *P* values are shown. (**E**) Reactivity of 4B7 with H7N9, PR8/felH7N2, and PR8/H7N3 viruses determined using immunofluorescence assay. MDCK cells were inoculated with the viruses or left non-inoculated. 4B7 was used as the first antibody in immunofluorescent assay. Scale bar, 100 µm. (**F**) Molecular docking of 4B7 scFv with H7N2-fel2016 HA. The structure of H7N2-fel2016 HA was modeled using SWISS-MODEL, and docking of the 4B7 scFv with the HA protein was performed using the ZDOCK server. (**G and H**) 4B7 footprint on the H7N2-fel2016 HA.

The G151E mutation in the antigenic site A mediated virus escape, indicating an important role of this domain in antibody-virus binding. To investigate conservation of this domain, the amino acid sequences of the H7 HA were aligned, and a phylogenetic tree was generated. A stark divergence could be observed between the HA genes of North American (NA) and Eurasian (EA) lineages, whereas the antigenic site A is a common and conserved motif in all H7 HA sequences (Fig. 3A). The amino acid sequence of the antigenic site A (148-RRSGSS-153) was found in the vast majority of EA lineage H7 HAs, while a small proportion of the isolates have 148-KRSGSS-153, such as H7N2-DK2007 used in this study (Fig. 3A). The 148-RRSGSS-153 sequence was found in half of NA lineage isolates, and the second-most commonly observed sequence is 148-TRSGSS-153 (Fig. 3A), which is found in the HA gene of A/feline/New York/16-040082-1/2016 (H7N2-fel2016). Therefore, although there is sequence variation, the antigenic site A, especially G151, targeted by the mAb 4B7 is highly conserved in the H7 subtype.

Subsequently, cross-reactivity of 4B7 with different influenza subtypes was examined. Reactivity of the antibody with the HA proteins of representative subtypes in group 1 (H1N1, H2N2, H5N1, and H9N2) and group 2 (H3N2, H4N6, H7N7, H7N9, and H15N8) was tested in ELISA, and 4B7 only reacted with H7N7 and H7N9 HAs (Fig. 3B). To investigate the cross-reactivity of 4B7 with different H7 viruses, 6:2 reassortant viruses based on the PR8 internal genes were generated (Table S4). The HA and NA genes of PR8/H7N4, PR8/H7N3, and PR8/felH7N2 viruses were derived from A/Chicken/Jiangsu/2018 (H7N4-JS2018), A/Mexico/InDRE7218/2012 (H7N3-Mex2012), and A/feline/New York/16-040082-1/2016 (H7N2-fel2016), respectively. H7N9-GD15, H7N4-JS2018, and H7N2-DK2007 represent EA lineage, and H7N2-fel2016 and H7N3-Mex2012 represent the NA lineage (Fig. 3A). There are high similarities of HA amino acid sequences within H7 viruses of EA (93.4-95.2%) and NA lineages (90.9%) and low identities ranging from 80.7% to 85.7% between the viruses of EA and NA lineages (Fig. S1B). Of note, the amino acid at position 151 in HA of these viruses is G, whereas residue 148 in H7N2-fel2016 and H7N2-DK2007 HAs is T and K, respectively (Fig. 3A). 4B7 had high VN activities against H7N9-GD15, PR8/H7N4, and PR8/H7N3 viruses, with minimal neutralizing concentrations (MNC) of 0.1, 0.01, and 0.06 µg/mL, respectively (Fig. 3C). Nevertheless, the antibody showed significantly lower VN titers against H7N2-DK2007 (MNC: 10 µg/mL) and undetectable neutralizing activity against PR8/felH7N2 at up to 100 µg/mL (Fig. 3C). Similar patterns were observed for HI activity of the antibody against H7 viruses (Fig. 3D). Additionally, the mAb 4B7 had reactivity with H7N9 and PR8/H7N3 in IFA, as expected, and unexpectedly, PR8/H7N2 was also recognized by the antibody (Fig. 3E). Interestingly, molecular docking analysis demonstrated that the 4B7 scFv did not interact with the antigenic site A of H7N2-fel2016 (Fig. 3F) but turned to bind a domain in the stalk distant from the antigenic site A (Fig. 3G and H). These findings suggest that the mAb 4B7 broadly reacts to diverse H7 viruses, and antibody reactivity with the viruses is affected by the variation of residue 148 in the antigenic site A.

### The mAb 4B7 protects mice from H7N9 virus infection in prophylactic and therapeutic settings via intraperitoneal administration

One potential use of anti-influenza mAb therapy is the prophylactic administration of antibodies to people who are likely exposed to infected humans or animals. To assess *in vivo* protection efficacy conferred by 4B7, an antibody transfer study was performed in mice in prophylactic and therapeutic settings. BALB/c mice were administered with the antibody at 30, 20, 10, and 5 mg/kg via the intraperitoneal (i.p.) route and then infected with 10 50% mouse lethal dose (MLD_50_) of the H7N9 maSDL124 virus through the intranasal (i.n.) route after 2 h (Fig. S2A). Body weight of the mice administrated with the antibody at all doses steadily increased (Fig. S2B), and administration of 4B7 completely protected against mortality (100% survival) (Fig. S2C). The PBS-inoculated mice started to lose body weight from day 3 post-challenge (p.c.) and all succumbed to infection at day 9 p.c. (Fig. S2B and C). At days 3 and 5 p.c., lung virus loads around 10^3.5^ 50% tissue culture infectious dose (TCID_50_)/0.1 mL were detected in the PBS-treated mice and were significantly decreased in the antibody-treated mice (Fig. S2D). At day 3 p.c., lung virus titers in the 4B7-treated mice were below the detection limit, and at day 5 p.c., a low amount of the virus was isolated from the lung of one mouse that received 30 mg/kg of the antibody (Fig. S2D).

In terms of pathology, no obvious lesions were observed in the lungs of the non-infected mock mice, while severe tissue lesions such as extensive infiltration of lymphocytes and neutrophils marked incrassation of the alveolar wall, and cellular necrosis was seen in the lungs of the PBS-inoculated mice (Fig. S2E). The antibody at all doses significantly reduced the severity of lung tissue damage compared to the PBS-inoculated mice (Fig. S2F and G), whereas mild incrassation of the alveolar wall, cellular necrosis, and infiltration of inflammatory cells were still observed in the mice treated with low doses (5 and 10 mg/kg) of 4B7 (Fig. S2E).

Subsequently, a therapeutic regimen was tested to simulate a clinical scenario of treatment of influenza virus infection in humans. Mice were infected with 10 MLD_50_ of maSDL124 12 h or 24 h before mAb administration (Fig. S3A and D). The PBS-treated mice lost their weight fast, and all died within 5 days p.c. (Fig. S3B, C, E and F). The mice received the antibody (5 and 10 mg/kg) at 12 h p.c. rapidly lost weight, and none survived beyond day 8 p.c. (Fig. S3B and C). Treatment with 4B7 at 20 and 30 mg/kg reduced weight loss, and the mice regained weight from day 9 p.c. (Fig. S3B), conferring 60% and 100% protection, respectively (Fig. S3C). When the antibody was administered 24 h after infection, all the mice that received 5 mg/kg of 4B7 quickly died, and the antibody at 10 and 20 mg/kg provided 20% protection. Antibody treatment at 30 mg/kg protected 40% of the mice (Fig. S3E and F).

Taken together, these findings indicated that the mAb 4B7 is prophylactically and therapeutically protective against H7N9 infection via systemic delivery, and an early administration of a high dose was required for a potent therapeutic protection.

### Intranasal administration of the mAb 4B7 is superior to intraperitoneal administration in a prophylactic setting

**Fig 4:**
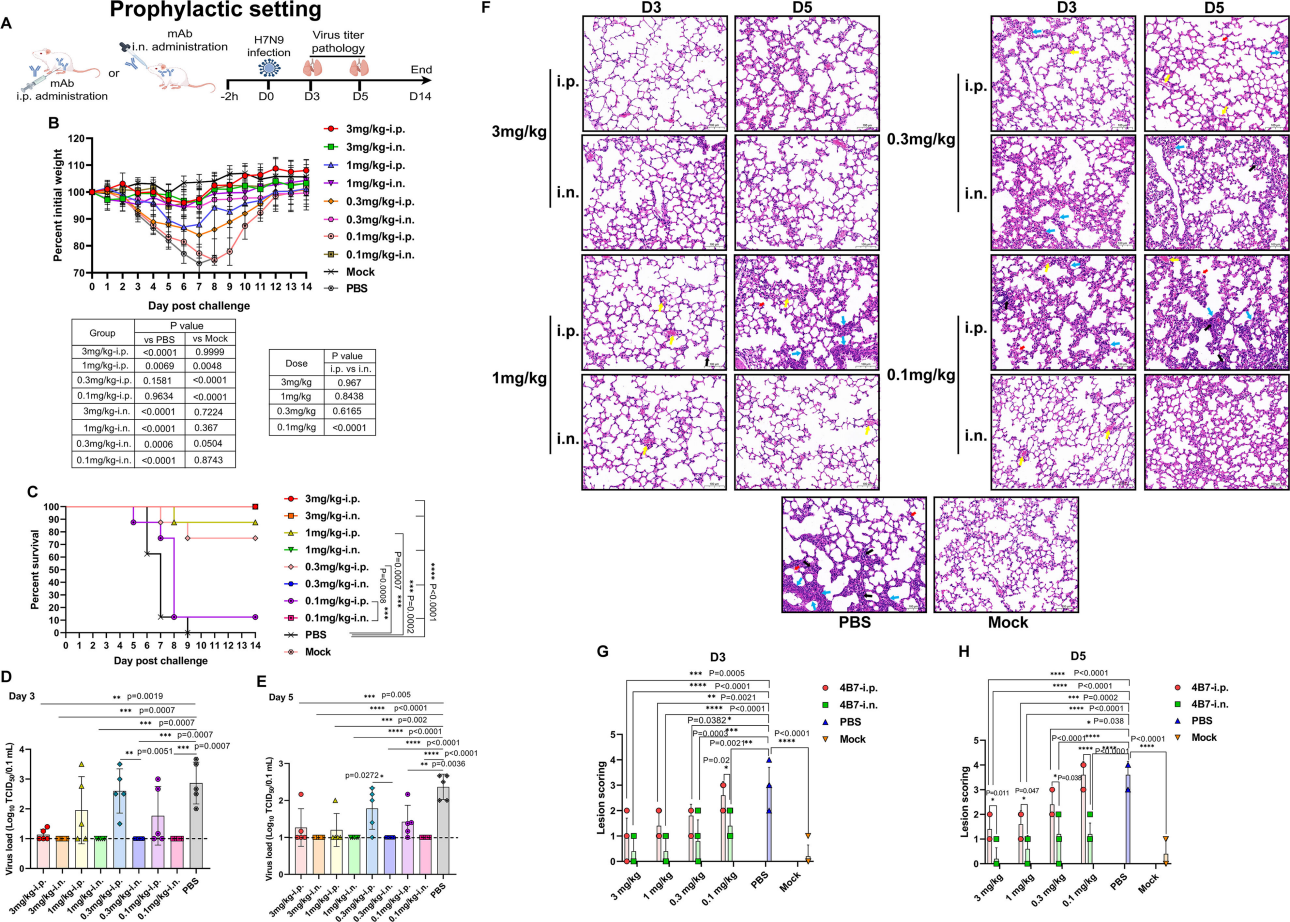
Prophylactic efficacy of the mAb 4B7 via intranasal and intraperitoneal administration. (**A**) Study design. Groups of mice (*n* = 18) were administered with the antibody at 3, 1, 0.3, and 0.1 mg/kg via the intraperitoneal (i.p.) or intranasal (i.n.) route or with PBS 2 h before infection with the H7N9 virus. Another group of mice (*n* = 8) was included as the mock control. Body weight (**B**) and survival (**C**) of the mice (*n* = 8). Virus titers in the lungs at days 3 (**D**) and 5 (**E**) post challenge. The lung tissues were collected from the virus-infected mice (*n* = 5), and virus titers (TCID_50_) were measured in MDCK cells. The dashed line indicates the detection limit of virus titration assay (10 TCID_50_/0.1 mL). Values below the detection limit were shown as 1 log_10_ TCID_50_/0.1 mL. (**F**) Lung pathology. The representative lung photomicrographs of each group are shown. The black arrows indicate infiltration of lymphocytes and neutrophils; the red arrows indicate cell debris in the lumen of bronchus; the blue arrows stand for incrassation of alveolar wall; the yellow arrows stand for accumulation of erythrocytes. Scale bar, 100 µm. (**G and H**) Scoring of lung lesions. Lesions were scored according to the following criteria: no or slight lesions, 0; mild lesions, 1; moderate lesions, 2; severe lesions, 3; very severe lesions, 4. Mean values ± standard error of the mean (SEM) of virus load and pathology (*n* = 5) or body weight (*n* = 8) per group were shown and analyzed using one-way ANOVA with Tukey’s multiple comparison test. Survival between the indicated two groups was analyzed using the Log-rank (Mantel-Cox) test. Asterisks indicate significant differences, and *P* values are shown.

Influenza virus infection is mainly restricted in the respiratory tract, and the efficacy of topically (i.n.) and systematically (i.p.) applied 4B7 against H7N9 virus was compared. Groups of mice were treated with the antibody at 3, 1, 0.3, and 0.1 mg/kg through the i.p. or i.n. route 2 h prior to infection (Fig. 4A). As shown in Fig. 4B, the PBS-inoculated mice exhibited dramatic weight loss after H7N9 virus infection, and all died at day 9 p.c. Body weight loss of the mice receiving the antibody at 3 and 1 mg/kg via the i.p. route was significantly reduced compared to that of the PBS group, whereas only i.p. administration of 4B7 at 3 mg/kg can significantly prevent weight loss compared to the mock group. Significant weight loss was detected in the mice treated with the antibody at 0.3 and 0.1 mg/kg via the i.p. route. In addition, i.n. administration of 4B7 at 3, 1, 0.3, and 0.1 mg/kg significantly reduced body weight loss compared to the PBS group, and there were no significant differences between the antibody-treated and mock control mice. When comparing the same doses, there was a significant difference in body weight between the mice treated with 0.1 mg/kg of 4B7 through the i.p. and i.n. routes. In terms of mortality, survival of the mice receiving the antibody at 3, 1, and 0.3 mg/kg via the i.p. route was significantly higher than that of the PBS group, and administration of 4B7 at 0.1 mg/kg protected 12.5% of the mice (Fig. 4C). By contrast, i.n.-administered antibody at all doses conferred complete protection against mortality caused by H7N9 virus infection (Fig. 4C). Moreover, at days 3 and 5 p.c., virus loads in the lungs of the mice receiving the antibody at 3 mg/kg through the i.p. route were significantly reduced compared to that of the PBS-treated mice (Fig. 4D and E). At day 5 p.c., i.p.-administered antibody at 1 and 0.1 mg/kg significantly decreased virus titers in the lungs (Fig. 4E). It is noted that the H7N9 virus could not be isolated from the lungs of the mice receiving the antibody at all doses via the i.n. route (Fig. 4D and E). Delivery of the antibody at all doses through the i.p. or i.n. route (except 0.1 mg/kg via the i.p. route) significantly alleviated lung pathology at day 3 and 5 p.c. (Fig. 4F through H).

Taken together, these results suggest that airway delivery of the mAb 4B7 significantly enhanced efficacy against H7N9 infection compared to systemic administration in a prophylactic setting.

### Intranasal delivery of 4B7 enhances therapeutic efficacy against H7N9 virus

**Fig 5:**
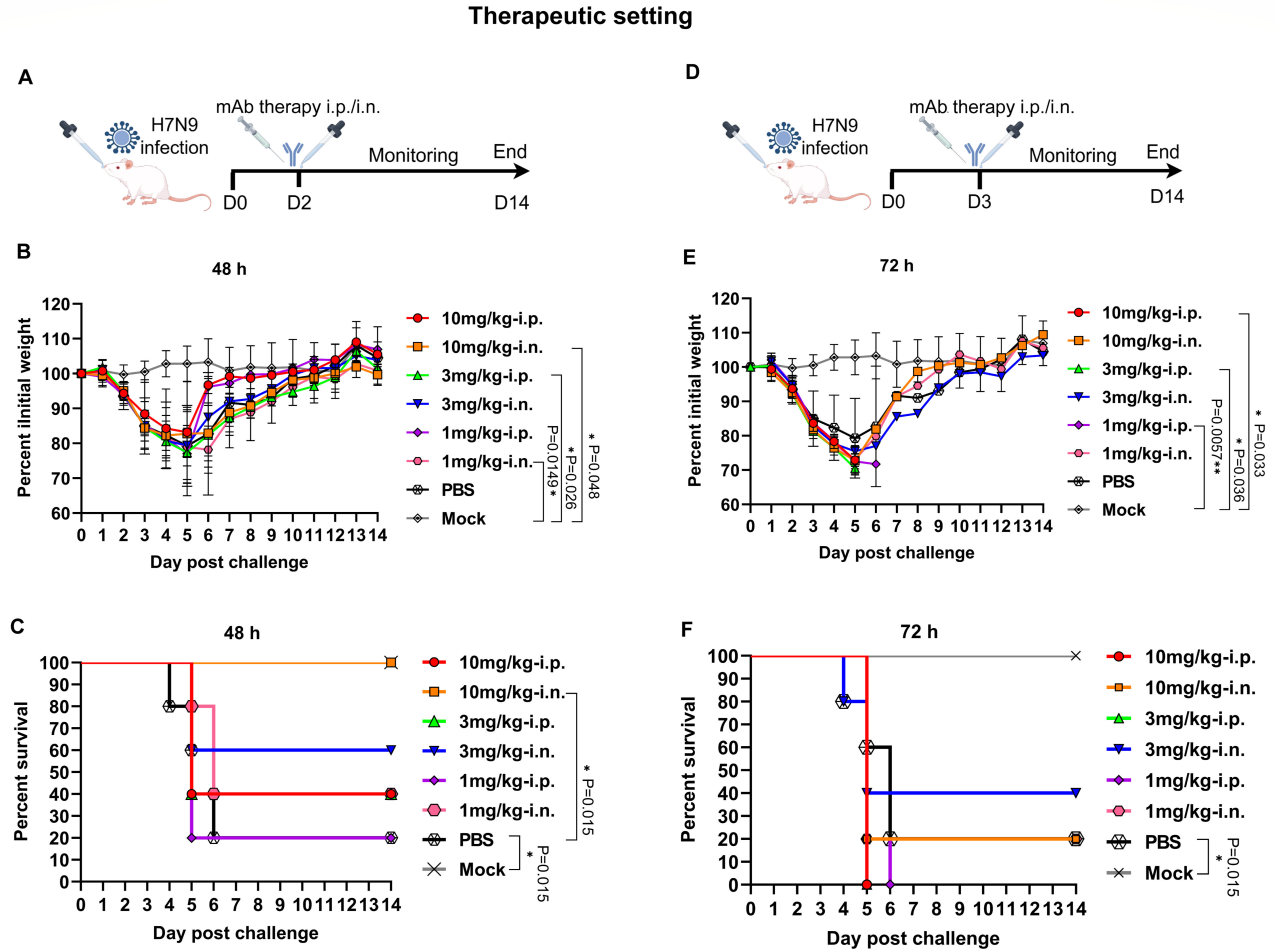
Therapeutic efficacy of the mAb 4B7 via intranasal and intraperitoneal administration. (**A and D**) Study design. Groups of mice (*n* = 5) were infected with 10 MLD_50_ of the H7N9 maSDL124 strain and then treated with the antibody at 10, 3, and 1 mg/kg via the intraperitoneal (i.p.) or intranasal (i.n.) route 48 h (**A**) or 72 h (**D**) later. Five mice were infected with maSDL124 and inoculated with PBS as the negative control. Another group of mice (*n* = 5) was used as the mock control. Body weight (**B and E**) and survival (**C and F**) of the mice were measured. Mean values ± standard error of the mean (SEM) of body weight per group are shown and analyzed using one-way ANOVA with Tukey’s multiple comparison test. Survival between the indicated two groups was analyzed using the log-rank (Mantel-Cox) test. Asterisks indicate significant differences, and *P* values are shown.

To compare the therapeutic efficacy of the mAb 4B7 delivered through the systemic or topical route, mice were infected with H7N9 virus and then treated with 4B7 via either the i.p. or i.n. route 48 and 72 h later (Fig. 5A and D). We found that body weight of all the infected mice gradually decreased from day 1 to 5 p.c. and was regained from day 6 p.c. (Fig. 5B and E). Compared to the PBS-inoculated mice, treatment with the antibody at all doses at 48 and 72 h post H7N9 infection failed to significantly reduce body weight loss (Fig. 5B and E). In addition, i.n. administration of 4B7 at 10 mg/kg at 48 h p.c. conferred complete protection, whereas 40% of the mice receiving the same dose via the i.p. route survived (Fig. 5C). When given at 72 h p.c., 10 mg/kg of the antibody conferred no protection (i.p.) or 20% protection (i.n.) against H7N9 virus infection (Fig. 5F). When the antibody dose was decreased to 3 mg/kg, i.n. administration at 48 h p.c. provided 60% protection, whereas a 40% survival was observed for the i.p. route (Fig. 5C). The antibody at 3 mg/kg administered at 72 h provided no (i.p.) or 40% protection (i.n.) against H7N9 virus infection (Fig. 5F). Moreover, i.n.-administered 4B7 at 1 mg/kg still protected 40% of the mice at 48 h, while i.p. delivery conferred 20% protection (Fig. 5C). The antibody at 1 mg/kg administered via the i.p. route provided no protection at 72 h, while i.n. delivery at the same dosage still protected 20% of the mice (Fig. 5F). These data demonstrated an enhanced therapeutic efficacy of locally administered 4B7 at 48 h post H7N9 virus infection.

### Humanized 4B7 sustains similar reactivity to H7 viruses as the murine 4B7

**Fig 6:**
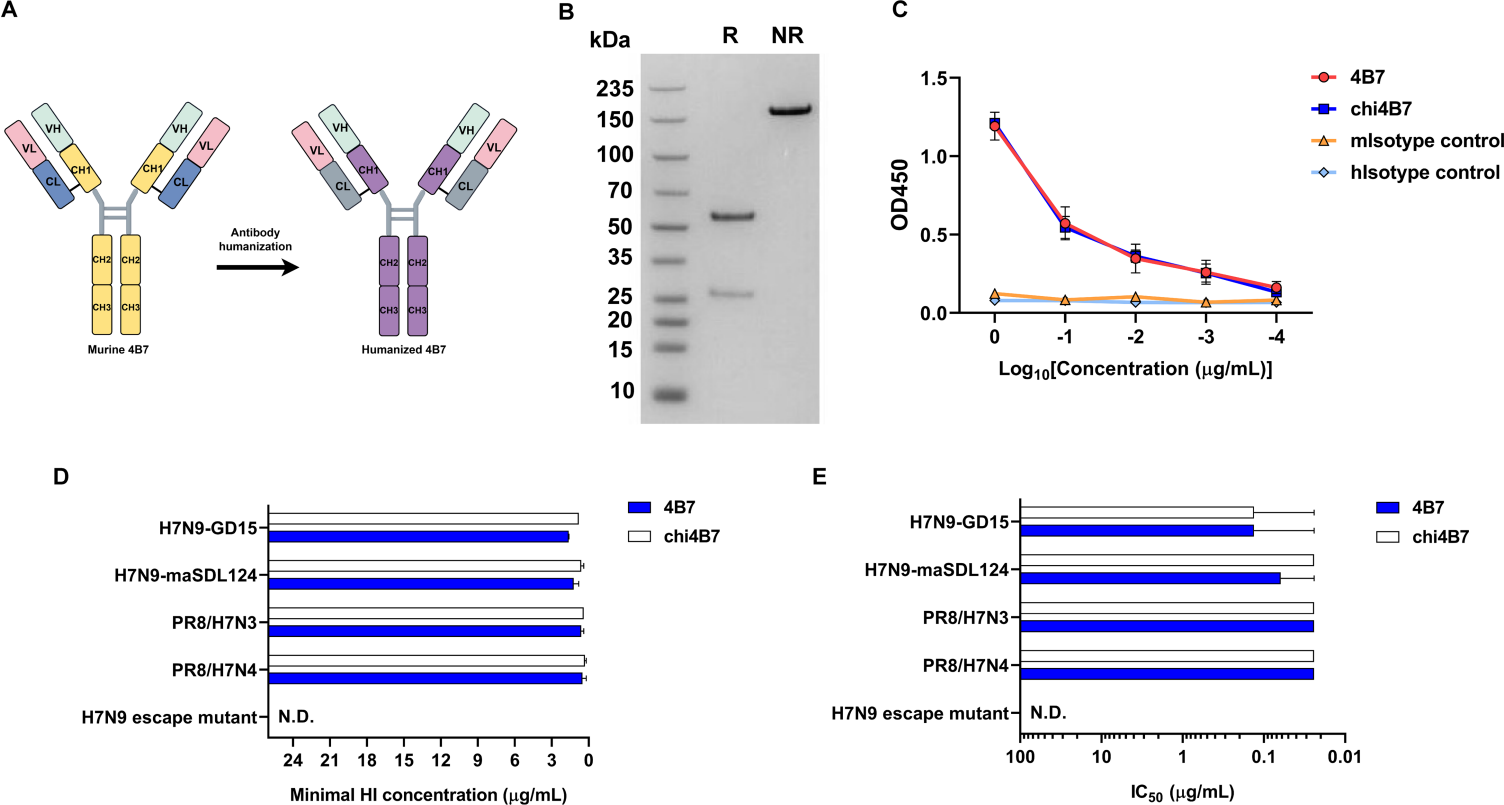
Generation and characterization of the humanized antibody. (**A**) Schematic illustration of antibody humanization. The variable heavy (VH) and variable light (VL) chains of the murine mAb 4B7 were grafted onto the human IgG1 backbone. (**B**) Electrophoresis analysis of the purified humanized antibody. R, reducing; NR, nonreducing. (**C**) Binding of the murine and humanized antibodies with the H7 HA protein determined using ELISA. Murine and human IgG1 antibodies were used as the isotype controls (mIsotype, murine IgG1; hIsotype, human IgG1). Chi4B7 indicates the humanized antibody. (**D**) Hemagglutination inhibition (HI) activities of the murine and humanized antibodies with different H7 viruses. Minimal HI concentration indicates the lowest antibody concentration required to inhibit the hemagglutination activity of the viruses. N.D., not detected. (**E**) Neutralizing activities of the murine and humanized antibodies with different H7 viruses. N.D., not detected. For ELISA, HI, and neutralization assays, mean values ± standard error of the mean (SEM) of at least two independent experiments are shown.

The goal of this study was to develop a neutralizing antibody for H7 influenza prevention and treatment in humans. For clinical use, the immunogenicity of murine antibodies should be diminished to reduce anti-antibody immune responses. To this end, humanization of the murine antibody 4B7 was performed. A chimeric antibody (chi4B7) was generated by grafting the VH and VL regions onto a human IgG1 and kappa backbone (Fig. 6A). The antibody chi4B7 was expressed in Chinese hamster ovary (CHO) cells and purified. The molecular sizes of the heavy chain and light chain were around 55 and 25 kDa, respectively, under reducing conditions and 150 kDa under non-reducing conditions (Fig. 6B). The H7 HA-binding, HI, and VN activities of chi4B7 against H7N9, PR8/H7N3, and PR8/H7N4 viruses were comparable to those of the parental mouse antibody (Fig. 6C through E). These findings suggest that humanization of the murine mAb 4B7 did not alter its activities with H7 viruses.

### The humanized antibody confers cross-protection against divergent H7 viruses

**Fig 7:**
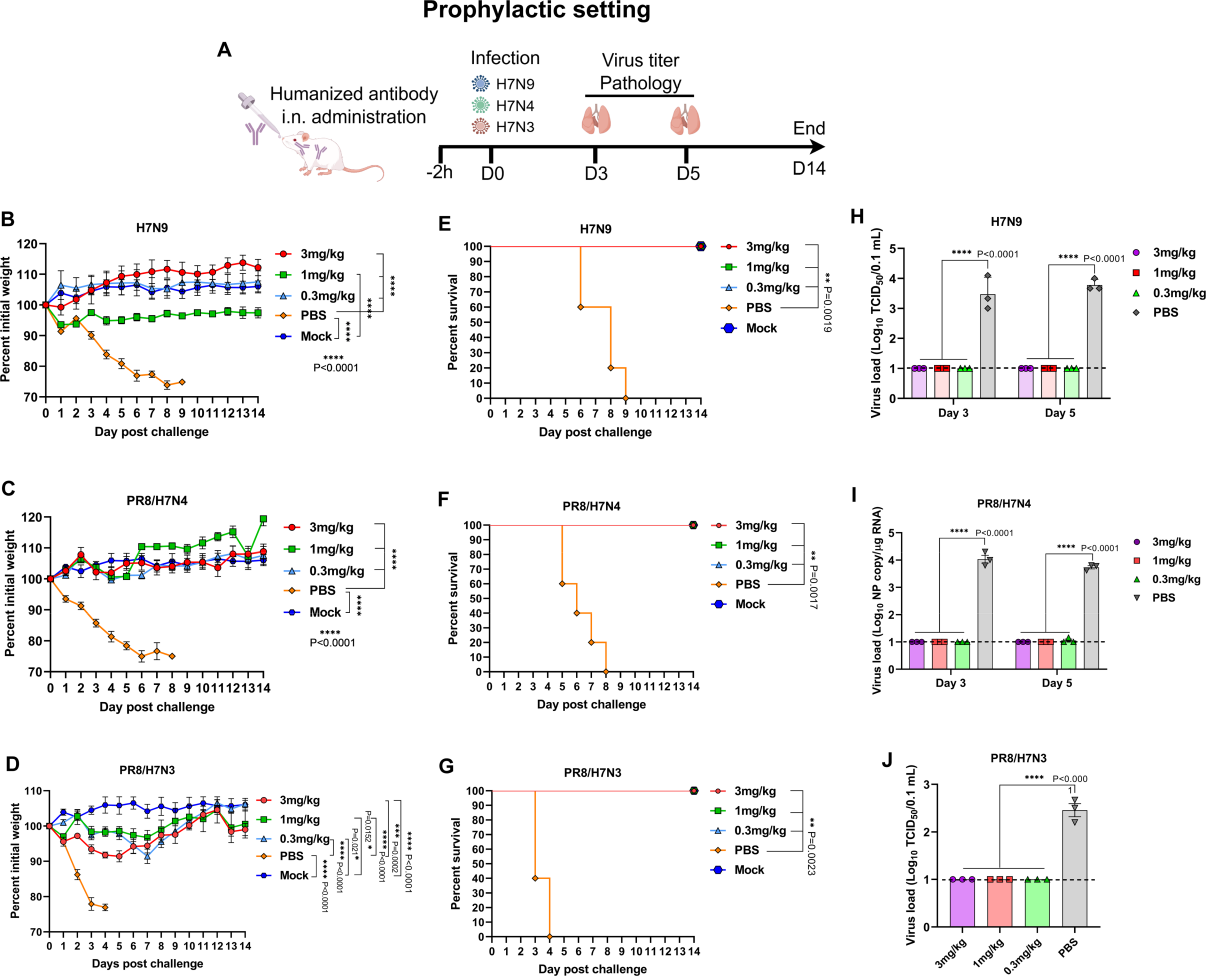
Prophylactic efficacy of the humanized antibody against diverse H7 viruses via intranasal administration. (**A**) Study design. Groups of mice (*n* = 11) were treated with the antibody at 3, 1, and 0.3 mg/kg or PBS via the intranasal (i.n.) route 2 h before infection with 10 MLD_50_ of the H7N9 (maSDL124), PR8/H7N4, and PR8/H7N3 viruses. Another five mice were used as the mock control. Body weight (**B, C, D**) and survival (**E, F, G**) of the mice. Virus titers (**H, I, J**) in the lungs of the virus-infected mice. The lung tissues were collected from the virus-infected mice (*n* = 3) at days 3 or 5 post-challenge and infectious virus titers (H7N9 and PR8/H7N3) or viral NP gene copies (PR8/H7N4) were measured. The dashed lines indicate the detection limit of virus titration assay (10 TCID_50_/0.1 mL or 10 copy/µg RNA). Values below the detection limit were shown as 1 log_10_ TCID_50_/0.1 mL or 1 log_10_ NP copy/µg RNA. Mean values ± standard error of the mean (SEM) of body weight (*n* = 5) or virus load (*n* = 3) per group are shown and analyzed using one-way ANOVA with Tukey’s multiple comparison test. Survival between the indicated two groups was analyzed using the log-rank (Mantel-Cox) test. Asterisks indicate significant differences, and *P* values are shown.

The humanized antibodies can broadly react to H7 viruses, and thus cross-protection provided by this antibody against different H7 viruses was assessed in mice in prophylactic and therapeutic settings. In a prophylaxis model, mice were i.n. administered with chi4B7 and then infected with H7N9, PR8/H7N4, and PR8/H7N3 viruses 2 h later (Fig. 7A). The PBS-inoculated mice rapidly lost their body weight after challenge, and all succumbed to virus infection (Fig. 7B through G). Body weight loss of all the mice receiving 3, 1, and 0.3 mg/kg of chi4B7 was significantly reduced compared to the PBS-inoculated mice after infection with H7N9, PR/H7N4, and PR8/H7N3 viruses (Fig. 7B through D). Compared to the mock control mice, there were no significant differences in the body weight of the antibody-treated mice post-infection with H7N9 and PR8/H7N4 viruses, except those receiving chi4B7 at 1 mg/kg after H7N9 virus infection, while significant weight loss was detected for the mice administered with the antibody at all doses after PR8/H7N3 challenge (Fig. 7B through D). It is noted that the humanized antibody completely conferred protection against mortality after infection with H7N9, PR8/H7N4, and PR8/H7N3 viruses (Fig. 7E through G). For virus load determination, because PR8/H7N4 had a low virus titer in MDCK cells (Table S4), the virus in the lungs could not be isolated in MDCK cells, and thus quantitative real-time PCR was used to measure lung virus loads of this virus. Lung virus loads of all antibody-treated mice were below the detection limit and significantly lower than that of the PBS-inoculated mice (Fig. 7H through J). Lung pathology caused by H7N9 and PR8/H7N4 viruses was significantly alleviated in the mice receiving 3 and 1 mg/kg of the antibody (Fig. S4 and S5). For the PR8/H7N3 virus, although the severity of lung lesions in the antibody-treated mice was significantly decreased, marked pathological changes including hemorrhage, infiltration of inflammatory cells, and incrassation of alveolar wall were still seen in the mice receiving the antibody (Fig. S6).

**Fig 8:**
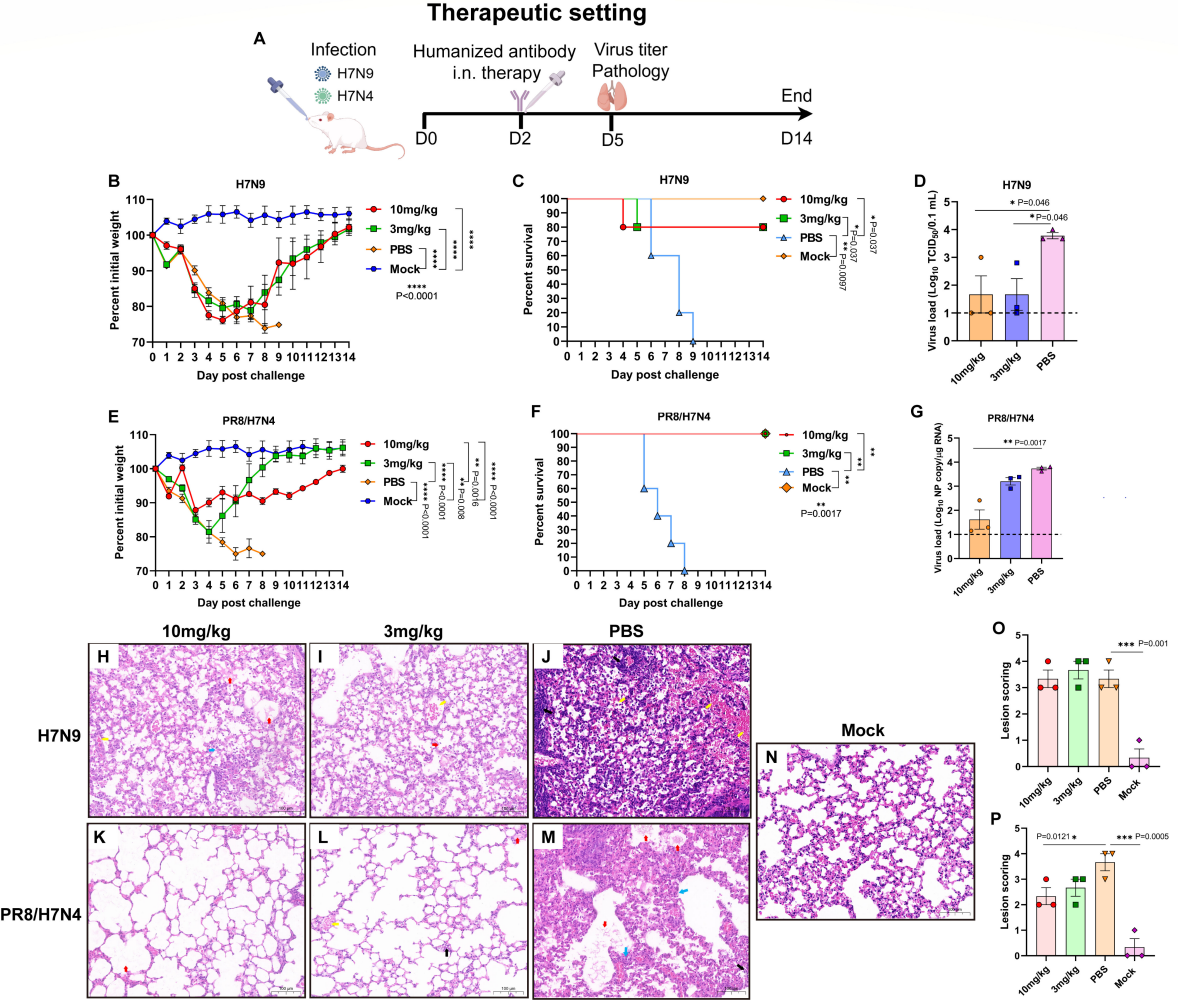
Therapeutic efficacy of the humanized antibody against different H7 viruses via intranasal administration. (**A**) Study design. Groups of mice (*n* = 8) were infected with the H7N9 (maSDL124) and PR8/H7N4 viruses and then treated with the antibody at 10 or 3 mg/kg or PBS via the intranasal (i.n.) route 48 h later. The PBS-treated and mock control mice were shared with the prophylactic study. Body weight (**B and E**) and survival (**C and F**) of the mice. Virus titers (**D and G**) in the lungs of the virus-infected mice at day 5 post-challenge. The lung tissues were collected from the virus-infected mice (*n* = 3), and infectious virus titers (H7N9) or viral NP gene copies (PR8/H7N4) were measured. The dashed lines indicate the detection limit of virus titration assay (10 TCID_50_/0.1 mL or 10 copy/µg RNA). Values below the detection limit were shown as 1 log_10_ TCID_50_/0.1 mL or 1 log_10_ NP copy/µg RNA. (**H–J**) Lung pathology of the chi4B7-treated mice after H7N9 virus infection. (**K–M**) Lung pathology of the chi4B7-treated mice after PR8/H7N4 virus infection. (**N**) Lung pathology of the mock mice. The black arrows indicate infiltration of lymphocytes and neutrophils; the red arrows indicate cell debris in the lumen of the bronchus; the blue arrows stand for incrassation of the alveolar wall; the yellow arrows stand for accumulation of erythrocytes. Scale bar, 100 µm. (**O and P**) Scoring of lung lesions. Lesions were scored according to the following criteria: no or slight lesions, 0; mild lesions, 1; moderate lesions, 2; severe lesions, 3; very severe lesions, 4. Mean values ± standard error of the mean (SEM) of body weight (*n* = 5) and virus load or pathology (*n* = 3) per group are shown and analyzed using one-way ANOVA with Tukey’s multiple comparison test. Survival between the indicated two groups was analyzed using the log-rank (Mantel-Cox) test. Asterisks stand for significant differences, and *P* values are shown.

To assess the therapeutic efficacy of the humanized antibody against different H7 viruses, mice were infected with H7 viruses and then treated with chi4B7 through the i.n. route 48 h later (Fig. 8A). The H7N9 subtype AIVs cause the largest number of human infection cases and are still prevalent in poultry (1, 6, 9). The H7N4 subtype AIV has caused a human infection in 2018 (10), and a recent study highlighted circulation of this subtype in shorebirds, their pathogenicity in mice without prior adaptation, and potential threat to public health (11). H7N9 and PR8/H7N4 viruses were selected for the therapeutic study for these reasons. Body weight of the virus-infected mice decreased, and those who received the antibody (10 and 3 mg/kg) at day 2 p.c. recovered shortly and gained weight on days 4 to 6 after treatment (Fig. 8B and E). One mouse succumbed to H7N9 infection at days 4 and 5 after receiving the antibody at 10 and 3 mg/kg, respectively. The humanized antibody at these two dosages conferred 80% protection against H7N9 virus infection (Fig. 8C). Antibody therapy also significantly reduced H7N9 virus titers in the lungs (Fig. 8D). In addition, chi4B7 treatment conferred complete protection against PR8/H7N4 virus infection (Fig. 8F), and lung virus loads were also significantly decreased after treatment with 10 mg/kg of the antibody (Fig. 8G). However, moderate-to-severe lung lesions were still observed in the mice therapeutically treated with the antibody at 10 and 3 mg/kg after infection with H7N9 and PR8/H7N4 viruses, despite that treatment with 10 mg/kg of the antibody significantly alleviated lung lesions in the PR8/H7N4-infected mice (Fig. 8H through Q).

These data showed that the humanized antibody conferred prophylactic and therapeutic cross-protection against divergent H7 viruses and reduced virus burden in the lungs, whereas antibody therapy did not significantly alleviate lung pathology.

### Airway-delivered humanized antibody has a long effective prophylaxis window

**Fig 9:**
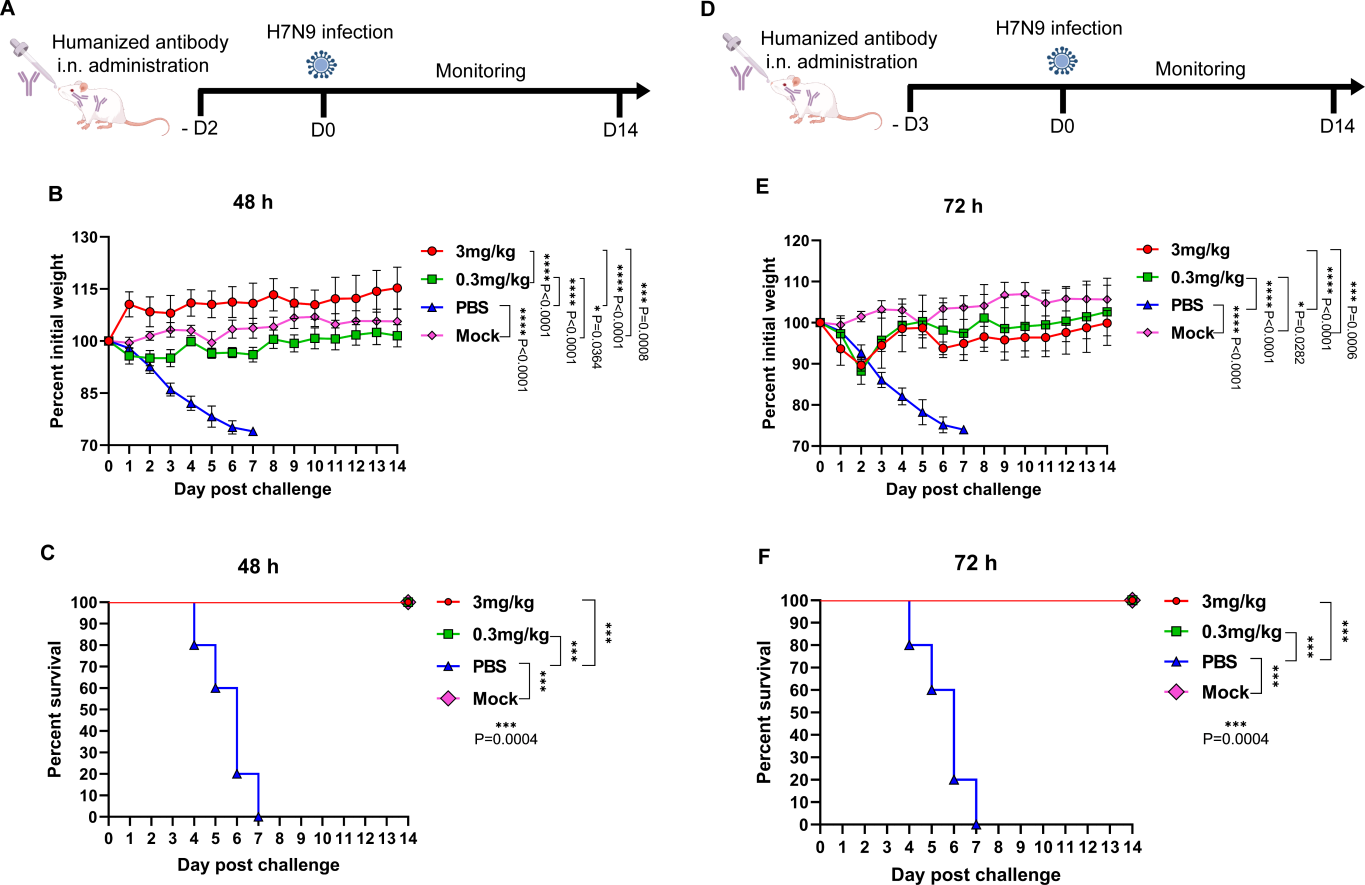
The effective prophylactic window of the humanized antibody. (**A and D**) Study design. Groups of mice (*n* = 7) were administered with the chi4B7 antibody at 3 and 0.3 mg/kg or PBS through the intranasal (i.n.) route 48 or 72 h before infection with 10 MLD_50_ of the H7N9 maSDL124 virus. Another group of mice (*n* = 5) was used as the mock control. Body weight (**B and E**) and survival (**C and F**) of the mice. Mean values ± standard error of the mean (SEM) of body weight (*n* = 5 or 7) per group are shown and analyzed using one-way ANOVA with Tukey’s multiple comparison test. Survival between the indicated two groups was analyzed using the log-rank (Mantel-Cox) test. Asterisks stand for significant differences, and *P* values are shown.

It is of interest to determine how long the effective prophylaxis window is for airway-delivered chi4B7. A survival study was performed in the H7N9 infection model with antibody administration initiated at 48 and 72 h before virus infection (Fig. 9A and D). The antibody at 3 and 0.3 mg/kg delivered at 48 and 72 h before infection significantly reduced body weight loss when compared to the PBS-inoculated mice, whereas only the 3 mg/kg dose given at 48 h prior to infection can confer protection against weight loss compared to the mock control mice (Fig. 9B and E). In addition, i.n. administration of the antibody at 3 and 0.3 mg/kg at 48 and 72 h before infection conferred full protection against mortality (Fig. 9C and F). These findings indicate that the humanized antibody has a long effective window when prophylactically delivered to the airway.

## DISCUSSION

In this study, a murine mAb against the HA of H7N9 virus was generated, and a humanized version of this antibody was produced. The antibody binds critical residues in the VED and RBS in H7 HA and particularly, the G151E mutation in the antigenic site A mediated virus escape from the antibody. The murine and humanized antibodies exhibited cross-binding, HI, and neutralizing activities with diverse H7 viruses. Airway delivery of the murine antibody reduced the dose required for potent prophylactic and therapeutic protection against H7N9 virus in mice compared to systemic administration. Intranasal administration of the humanized antibody conferred broad protection against diverse H7 viruses and had a long effective prophylaxis window. Our findings suggest that airway delivery of the humanized antibody is a feasible and cost-effective approach for prevention and therapy against H7 influenza.

Many mAbs against the H7N9 subtype AIV were generated to develop novel antiviral agents, and systemic administration of antibodies can protect from overt clinical signs and mortality caused by H7N9 viruses (23, 25, 32). Respiratory virus infection, including influenza infection, in humans is typically initiated and limited in the respiratory tract (17, 33). Thus, concentrations of antibodies in the site of infection are essential for protection. However, the bioavailability of systemically administered antibodies in the respiratory tract is low, and high antibody doses must be administered for protection against fatal infection (28). This provides a rationale for delivering anti-influenza antibodies directly to the airway side of the respiratory tract.

Consistent with this idea, we found that intranasal administration of the mAb 4B7 via the i.n. route improved prophylactic efficacy against H7N9 virus infection in mice compared to the i.p. route. Particularly, 3 mg/kg of the antibody administered via the i.p. route was required for significant protection against body weight loss and mortality, whereas i.n. delivery of 4B7 at 0.1 mg/kg was sufficient to confer protection. In addition, i.n. delivery also enhanced the therapeutic efficacy of the antibody against H7N9 virus. Previous studies showed that compared to systemic administration, airway delivery enhanced the efficacy of antibodies against respiratory pathogens, including influenza virus, respiratory syncytial virus, and SARS-CoV-2 (27, 28, 34, 35). In this regard, our findings correlate with these reports, highlighting the advantage of airway delivery over systemic administration of antibodies in treating respiratory infections. Besides enhancing efficacy, airway delivery that allows dose sparing is a highly desirable approach to reduce the cost and increase the accessibility of antibody therapy for large-scale administration. Although airway delivery of the mAb 4B7 is superior to systemic administration in mice, it is still necessary to compare the efficacy of the antibody delivered via these two routes in ferrets, the optimal disease model for human influenza. Friesen et al. demonstrated prophylactic and therapeutic efficacy of a human mAb CR6261 at 30 mg/kg against H5N1 subtype AIV in ferrets through intravenous injection (36). Another study showed that i.p. administration of MEDI8852 at 25 mg/kg, a broadly reactive mAb against the HA stem, was effective for lethal H5N1 and H7N9 infection and can protect naive ferrets from airborne transmission of H1N1pdm09 (37). Therefore, head-to-head comparison studies are required to determine whether airway delivery of anti-influenza antibodies outperforms systemic administration in ferrets.

Our study also presented new findings supporting antibody airway delivery for zoonotic influenza treatment. Previous reports on mAb delivery are mainly focused on seasonal influenza, including H1N1, H1N1pdm09, and H3N2 viruses. Of particular note, human infections with zoonotic H7 viruses have distinct clinical manifestations compared to seasonal influenza. H7N9 infection has usually presented with severe viral pneumonia, and some cases were complicated by ARDS and multiorgan failure. Unlike patients with seasonal or H5N1 influenza, patients with H7N9 disease were more likely to be older and have underlying comorbidities. Similar clinical features were also observed for the H7N4 human infection case. Therefore, it is essential to assess the efficacy of anti-H7 antibodies administered through airway delivery. A previous report analyzed the efficacy of a neutralizing antibody (Mab 62) against the H7N7 subtype AIV via the i.n. and i.p. routes (26), while our study presented more systemic findings compared to that study. First, the mAb 4B7 and humanized antibody were assessed in a more clinically relevant manner. The current standard-of-care anti-influenza therapy, oseltamivir, is usually administered 48 h post-exposure or post-infection. The Mab 62 was administered through the i.p. or i.n. route at 24 h before H7N7 virus infection, whereas the efficacy of i.n. delivery of the humanized 4B7 at 48 and 72 h prior to H7N9 infection was determined. In a therapeutic setting, one dose of the Mab 62 was given at 24 h after H7N7 virus infection, and the efficacy of the murine and humanized 4B7 was assessed at 48 and 72 h p.c. The humanized antibody administered via the i.n. route at 72 h (0.3 mg/kg) before infection or 48 h (10 mg/kg) post infection provided good protection, indicating its potential as antiviral prophylaxis and therapy against H7 influenza infection. Moreover, the Mab 62 recognizes a highly conserved epitope (K715) in H7 HAs and has cross-HI and VN activities with divergent H7 viruses, whereas cross-protection against H7 viruses was not determined. In this study, the humanized antibody chi4B7 conferred cross-protection against infection with different H7 viruses in mice, highlighting its potential to be used as a broad antiviral agent against H7 viruses.

For passive antibody therapy, the immunogenicity of murine antibodies in humans should be minimized to reduce potential side effects. Antibody humanization is a common approach for this purpose, and it is critical to preserve antigen-binding activities in this process. Herein, grafting the variable regions onto a human IgG1 backbone was performed for 4B7 humanization, causing no alterations in antigen binding, HI, and VN activities of the antibody. More importantly, the humanized antibody provided prophylactic and therapeutic protection against various H7 viruses via i.n. administration. Nevertheless, the antibody generated through variable region grafting is not a full humanized antibody. To further reduce anti-drug antibodies against murine antibody components, new humanization methods, including CDR grafting, specificity-determining residues grafting, or framework (FR) shuffling, can be employed (38). However, antigen affinity of the humanized antibodies generated with these strategies should be carefully evaluated due to incompatibility between human FRs and mouse CDRs.

The mAb 4B7 footprint covers crucial domains in the RBS (130-loop and 150-loop) and a major antigenic region (site A). The antigenic site A is highly conserved in H7 HAs and is a dominant target of neutralizing antibodies. Interestingly, the G151E mutation in the antigenic site A mediated H7N9 virus escape from 4B7 neutralization. Mutation at G151 is also associated with immune escape of H7N9 viruses from other neutralizing antibodies (30, 39–41). A recent report revealed that an H7N9 field isolate (SD001) with a natural G151E variation in the HA can escape from two anti-H7N9 neutralizing antibodies, strengthening the critical role of G151 in affecting antibody neutralization (32). Moreover, variations in other residues in the antigenic site A also impact the reactivity of antibodies with H7 HA. The mAb 4B7 showed significantly lower neutralizing and HI activities against an EA lineage isolate H7N2-DK2007 with K148 and undetectable neutralizing and HI activities against an NA lineage isolate PR8/felH7N2 with T148. Intriguingly, the reactivity of the antibody with PR8/felH7N2 was detected in IFA, which supported 4B7-H7N2 HA interaction in molecular docking. It is noted that 4B7 turned to recognize a new epitope in the HA stalk due to the presence of T148 in the antigenic site A. Epitope switching may lead to reduced neutralizing and HI activities but no impacts on HA binding. However, further experiments are required to verify the docking results, and it is interesting to investigate why mutations in residues 151 and 148 exert distinctive effects on 4B7 activities.

Many previously reported murine and human anti-H7 mAbs select escape mutant viruses with mutations at five key residues in the antigenic site A (25, 30, 41–44). In terms of neutralizing mechanisms of 4B7, the antibody processes HI activity against H7 viruses, indicating that it can block virus attachment to cell receptors. This may be associated with recognition of the 130- and 150-loop in the RBS. Of note, although the antigenic site A is outside the RBS, antibodies targeting this region can also inhibit virus binding to the receptors. A pan-H7 human mAb (rH7-235) recognizes residues in the antigenic site A outside of the RBS but near the edge of the RBS (45). rH7-235 IgG potently neutralizes H7N9 viruses and protects against H7N9 infection due to avidity effect and Fc steric hindrance. However, the role of critical domains targeted by 4B7 in virus neutralizing activity and protection needs to be determined by further studies. Therefore, variation of the five residues in the antigenic site A may affect activities of anti-H7 antibodies, and antibodies targeting this region may neutralize H7 viruses through distinct mechanisms, informing the rational design of therapeutics and vaccines against the H7 subtype influenza viruses.

Currently, the wide spreading of the H5N1 subtype AIV (clade 2.3.4.4b) worldwide and its spillover to mammals and humans poses a great challenge to public health. Besides the H5 subtype, avian influenza outbreaks caused by different H7 subtypes have occurred in five countries in the last century, and a total of 1,687 human cases with H7 influenza virus infection have been documented from 1959 to 2019 (1). Recently, several poultry outbreaks associated with highly pathogenic H7 subtype AIVs were reported in different countries, highlighting the pandemic potential of the H7 subtype AIVs (7, 8). The murine and humanized antibodies reported herein target conserved epitopes in the H7 subtype, which afford cross-reactivity of the antibodies with multiple H7 viruses. More importantly, the humanized 4B7 conferred broad protection against diverse H7 viruses when administered via the i.n. route. Therefore, the humanized antibody can serve as a broad antiviral therapy against the H7 subtype influenza.

In conclusion, we generated and characterized murine and humanized antibodies with cross-H7 binding, HI, and neutralizing activities. Airway delivery of the humanized antibody provided broad prophylactic and therapeutic protection against diverse H7 viruses. These features render the humanized antibody a favorable candidate for prophylaxis and therapy against the H7 subtype influenza. Moreover, epitope profiling of the antibodies also presented novel insights into understanding antibody response to H7 viruses and for vaccine design.

## MATERIALS AND METHODS

### Viruses, plasmids, proteins, and cells

The mouse-adapted H7N9 subtype AIV strain maSDL124 derived from A/chicken/Shandong/SDL124/2015 (Genbank accession numbers: MW397099 to MW397114) and the reassortant H7N9 virus GD15 derived from a highly pathogenic strain (A/chicken/Guangdong/GD15/2016) (EPI_ISL_305597) were generated previously (46, 47). The H7N2 subtype AIV of duck origin (H7N2-DK2007) was reported previously (48) and obtained from Prof. Liping Yan (Key Animal Virology Laboratories of the Ministry of Agriculture and Rural Affairs of China). The viruses were propagated in 10-day-old specific pathogen-free (SPF) embryonated chicken eggs (ECEs). The plasmids encoding the six internal proteins of PR8 were used for generation of the reassortant influenza viruses. The HA protein of the GD15 strain was expressed in the baculovirus expression system (31). Madin-Darby canine kidney (MDCK) cells and human embryonic kidney 293T cells were cultured in Dulbecco’s modified Eagle medium (DMEM) (ThermoFisher Scientific, Waltham, MA, USA) supplemented with 10% fetal bovine serum (FBS) (LONSERA, Suzhou, China) at 37°C with 5% CO2. CHO cells were cultured in DMEM/F12 medium (ThermoFisher Scientific) supplemented with 10% FBS at 37°C with 5% CO2.

### MAb screening and generation

MAbs against the H7N9 HA were generated using the hybridoma method previously, but only one antibody with a low VN titer was obtained (31). In that study, a total of 8 mice were immunized with the HA protein, and spleen cells were collected for fusion. The hybridomas derived from 5 mice were screened, and the remaining hybridomas were stored in liquid nitrogen. Herein, the frozen hybridomas were recovered and screened using ELISA and microneutralization assay. The positive clones were then subcloned using the limiting dilution method. Positive hybridomas were intraperitoneally injected into BALB/c mice. Ascites was collected, and antibody purification was performed using protein G affinity chromatography (GE Healthcare, Piscataway, NJ, USA).

### ELISA

ELISA was performed as previously reported (49). The HA protein (0.25 µg/mL) or the H7N9 viruses at 32 hemagglutination units (HAU) was immobilized onto 96-well plates in a carbonate buffer and incubated at 4°C overnight. The plates were washed three times with PBST (PBS with 0.05% Tween-20) and then blocked with 200 µL of 5% (wt/vol) skim milk in PBST at 37°C for 1 h. The plates were washed three times with PBST and then incubated with the antibodies at serial concentrations at 37°C for 1 h (100 µL/well). After washing, 100 µL of horseradish peroxidase-conjugated goat anti-mouse or -human IgG antibodies (1:5,000) was added and incubated for 1 h at 37°C. The plates were washed with PBST three times and developed with 100 µL of 3,3’,5,5’-tetramethylbenzidine substrate at room temperature (RT) for 15 min. Then, 50 µL of 3 M HCl was added to stop the reaction, and the absorbance at 450 nm was measured.

### HI test

HI tests were conducted according to the protocol of World Organization of Animal Health with some modifications. In brief, the antibodies were initially diluted by 100-fold, followed by 2-fold serial dilutions. The H7 viruses (4 HAU) were used as the antigens. HI titers were defined as the minimal concentrations of the antibodies required to completely inhibit the hemagglutination activity of the viruses.

### Microneutralization assay

MDCK cells at a density of 30,000 were seeded onto 96-well plates and cultured overnight to reach a confluency of 80%. The antibodies were 10-fold serially diluted and incubated with 100 TCID_50_ of H7 viruses at 37°C for 1 h. DMEM containing 1 µg/ml of tosyl-sulfonyl phenylalanyl chloromethyl ketone (TPCK)-treated trypsin was used for dilution of the viruses and antibody. The antibody-virus mixtures were transferred to MDCK cells and cultured for 48 h. The supernatants were then collected, and virus infection was determined using the hemagglutination test. The IC_50_ was calculated using nonlinear regression analysis with the GraphPad Prism (GraphPad Software, San Diego, CA).

### Antibody sequencing and molecular docking

RNA was extracted from the 4B7 hybridomas and then transcribed into cDNA. Antibody VH and VL genes were amplified using PCR and sequenced. Genetics composition of the antibody 4B7 was analyzed using the IMGT/V-QUEST program (https://www.imgt.org/IMGT_vquest/input). Homology models of the 4B7 scFv and the H7 HA protein structures were generated using the ABodyBuilder-ML server (https://opig.stats.ox.ac.uk/webapps/sabdab-sabpred/sabpred/abodybuilder/) and SWISS-MODEL server (https://swissmodel.expasy.org), respectively. Molecular docking of the 4B7 scFv and the HA proteins was performed using the ZDOCK server (https://zdock.wenglab.org/), and the complex was visualized and handled using the PyMOL software. The interaction surface of the scFv-HA complex was analyzed on the web PISA server (https://www.ebi.ac.uk/pdbe/pisa/).

### Escape mutant studies

To identify the epitope recognized by 4B7, escape mutants derived from the H7N9 GD15 strain were generated as reported previously (50). Briefly, 10^6^ 50% embryo infectious dose (EID_50_) of the virus in 0.5 mL was mixed with the antibody at 20 µg/mL and was incubated at RT for 1 h. Then, 0.1 mL of the mixture was inoculated into 10-day-old SPF ECEs. After incubation for 4 days, the allantoic fluids were collected for hemagglutination assay. These procedures were repeated twice by incubating serial dilutions (10^5^ to 10^8^) of the allantoic fluids with the antibody. Subsequently, HI titers of the antibody against the allantoic fluids were measured, and the viruses were defined as the escape mutants when HI titers of the mAb reduced by at least 8-fold compared to that against the parental virus. The HA gene of the escape mutants was sequenced.

### Generation of the reassortant H7 viruses

To validate the results of escape mutant studies, the reassortant H7N9 viruses carrying the G151E and I335V mutations in HA were generated. In addition, the HA and NA genes of the H7N9 (A/Guangdong/17SF003/2016, SF003) (EPI_ISL_280902), H7N2 (A/feline/New York/16-040082-1/2016, fel2016) (EPI_ISL_260817), H7N3 (A/Mexico/InDRE7218/2012, Mex2012) (EPI_ISL_128317), and H7N4 (A/chicken/Jiangsu/1/2018, JS2018) (EPI_ISL_332358) viruses were synthesized and cloned into the pHW2000 vector. Notably, none of the HA sequences harbored a polybasic cleavage site. The 6:2 reassortant viruses based on the PR8 internal genes were generated using reverse genetics.

For virus rescue, co-cultures of MDCK and 293T cells were co-transfected with the plasmids. These plasmids contained the HA and NA genes, along with six internal genes of PR8 (400 ng of each plasmid). The *Trans*IT-X2 Dynamic Delivery System (Mirus, Madison, WI, USA) was used for transfection purposes. At 24 h post-transfection, TPCK-treated trypsin (1 µg/mL) was added to the cell culture. At day 3 post-transfection, both the cells and supernatants were collected. Subsequently, 0.3 mL of this mixture was subjected to freeze-thaw cycles and then inoculated into 10-day-old SPF ECEs. The presence of the virus was detected using the hemagglutination assay.

### Pathogenicity of the reassortant H7 viruses in a PR8 backbone in mice

Morbidity and mortality of the rescued reassortant H7 viruses in the PR8 backbone in mice were determined. Groups of 6- to 8-week-old female BALB/c mice (*n* = 5) were intranasally inoculated with the PR8/H7N3 and PR8/H7N4 viruses at 10^6.0^, 10^5.0^, 10^4.0^, 10^3.0^, and 10^2.0^ EID_50_. Another group of mice (*n* = 5) was inoculated with PBS as the non-infected mock control. Clinical signs and body weight were monitored daily for 14 days, and mice that lost 25% or more of their initial body weight were euthanized and scored dead. The MLD_50_ was calculated.

### Prophylactic and therapeutic efficacy of the mAb 4B7 against H7N9 virus in mice via intraperitoneal administration

Passive transfer studies were performed to investigate the prophylactic and therapeutic efficacy of the mAb 4B7 in mice. To evaluate the prophylactic efficacy, 6- to 8-week-old female BALB/c mice (*n* = 11) were inoculated with the antibody at 30, 20, 10, and 5 mg/kg via the i.p. route. Another group of mice (*n* = 11) was inoculated with PBS as the negative control. Naïve mice (*n* = 5) were used as the non-infected mock control. After 2 h, the mice were anesthetized by i.p. injection with 0.1 mL of pentobarbital sodium and then inoculated with 10 MLD_50_ of maSDL124 virus via the i.n. route. Body weight was monitored daily for 14 days, and mice that lost 25% or more of their initial body weight were euthanized and scored dead. At days 3 and 5 p.c., three mice from the virus-infected groups were euthanized, and the lungs were collected for virus titration and histopathologic examination.

Moreover, in a therapeutic setting, 6- to 8-week-old female BALB/c mice (*n* = 5) were inoculated with 10 MLD_50_ of maSDL124 through the i.n. route and then treated with the antibody at 30, 20, 10, and 5 mg/kg at 12 and 24 h p.c. via the i.p. administration. The mice were monitored daily for 14 days, and weight loss and survival were recorded.

### Prophylactic and therapeutic efficacy of the mAb 4B7 against H7N9 virus in mice via intranasal administration

For prophylactic studies, the antibody at 3, 1, 0.3, and 0.1 mg/kg was administered to 6- to 8-week-old female BALB/c mice (*n* = 18) via the i.p. route for systematic administration or via the i.n. route for topical administration. Another group of mice (*n* = 18) was inoculated with PBS as the negative control, and eight naïve mice were used as the non-infected mock control. After 2 h, the mice were anesthetized and then infected with 10 MLD_50_ of maSDL124. Weight loss and survival of the mice were monitored for 14 days. At days 3 and 5 p.c., 5 mice from the virus-infected groups were euthanized, and the lungs were harvested for virus titration and histopathologic analysis. In a therapeutic setting, groups of mice (*n* = 5) were infected with 10 MLD_50_ of maSDL124 and then treated with 4B7 at 10, 3, and 1 mg/kg via the i.p. or i.n. route at 48 and 72 h p.c. Body weight and survival were monitored for 14 days.

### Expression and characterization of the humanized antibody chi4B7

To generate the humanized antibody, the VH and VL segments of the mAb 4B7 were PCR-amplified and then individually cloned into the pCDNA3.4 expression vector encoding human IgG1 constant regions. The heavy- and light-chain expression plasmids were co-transfected into CHO cells. At day 7 post-transfection, the supernatants were harvested and centrifuged at 5,000 rpm for 20 min, followed by filtration through 0.45 µm filter apparatus to remove remaining cell debris. Antibodies were purified from the supernatants using Protein G columns (GE Healthcare). The concentration of the purified antibody was measured, and the antibody was assessed by sodium dodecyl sulfate polyacrylamide gel electrophoresis under the reducing and non-reducing conditions. In addition, binding, HI, and VN activities with H7 viruses of the humanized antibody were measured.

### Prophylactic and therapeutic efficacy of the humanized antibody against divergent H7 viruses in mice

Protective efficacy of the humanized antibody (chi4B7) against different H7 viruses was assessed in mice in prophylactic and therapeutic settings. In prophylactic studies, groups of 6- to 8-week-old female BALB/c mice (*n* = 11) were administered with chi4B7 at 3, 1, and 0.3 mg/kg via the i.n. route. Another group of mice (*n* = 11) was inoculated with PBS as the negative control, and five naïve mice were used as the non-infected mock control. The mice were inoculated with 10 MLD_50_ of the maSDL124, PR8/H7N4, and PR8/H7N3 viruses 2 h after antibody administration. Weight loss and survival were monitored for 14 days. At days 3 and 5 p.c., 3 mice from the virus-infected groups were euthanized, and the lungs were harvested for virus load measurement and histopathologic analysis. For therapeutic experiments, groups of mice (*n* = 8) were first i.n. inoculated with 10 MLD_50_ of the maSDL124 and PR8/H7N4 viruses and administered with chi4B7 at 10 and 3 mg/kg through the i.n. route 48 h later. Body weight and survival of the mice were monitored for 14 days. At day 5 p.c., 3 mice from the virus-infected groups were euthanized, and the lungs were collected for virus titration and histopathologic examination. The data of the mock control, virus load, and lung pathology at day 5 p.c. of the PBS-inoculated mice were shared by the prophylactic and therapeutic experiments. In addition, to assess the effective prophylactic window of airway-delivered chi4B7, groups of mice (*n* = 7) were i.n. administered with the antibody at 3 and 0.3 mg/kg. Another group of mice (*n* = 7) was inoculated with PBS as the negative control, and five naïve mice were used as the non-infected mock control. The mice were infected with the H7N9 maSDL124 virus at 48 or 72 h post antibody administration, and body weight and survival were monitored for 14 days.

### Quantitative real-time PCR

Since PR8/H7N4 had a low virus titer in MDCK cells (Table S4) and virus in the lung samples was not isolated in MDCK cells, quantitative real-time PCR was used to measure NP gene copies in the lungs. A plasmid containing the PR8 NP gene was used as the standard. The standard plasmid was serially diluted, and the samples containing the NP gene at 10^1^–10^7^ copies/µL were used in PCRs to generate the standard curve. Total RNA was extracted from the tissue samples using TRIzol reagent (Vazyme) and then reverse-transcribed into cDNA using the HiFiScript SuperFast gDNA Removal cDNA Synthesis Kit (CWBIO, Taizhou, China). The system of PCR was composed of 1 µL of the tissue cDNA or the standards, 0.4 µL of the primers, 0.2 µL of the probe, and 10 µL of 2 × AceQ qPCR Probe Master Mix (Vazyme). The sequences of the primers and probe were as follows: forward primer, 5′-AAGGTGGTCCCAAGAGGGA-3′; reverse primer, 5′-GCTGCCATAACGGTTGTTCTG-3′; probe, FAM-TACAGAGAAATCT-MGB. PCRs were conducted using the CFX Connect Real-Time System with the following cycles: one cycle for denaturing at 95^°^C for 5 min, 40 cycles for PCR at 95^°^C for 10 s and 60^°^C for 30 s. The standard curve was generated using the Bio-Rad CFX Maestro software, and the NP gene copies were calculated based on the standard curve.

### Statistical analysis

For ELISA, HI, and VN assays, mean values ± standard error of the mean (SEM) of at least two independent experiments were shown. For body weight and survival, mean values ± SEM of at least five mice per group were shown. Virus loads and lung lesion scores of three or five individual animals were shown as mean values ± SEM. The data were analyzed using one-way ANOVA in GraphPad Prism software. Multiple comparisons were conducted by comparing the mean of each group with every other group using Tukey’s test for multiple comparison correction.

## Data Availability

Reasonable requests for the data and materials can be made to the authors.

## SUPPLEMENTAL MATERIAL

Supplemental figures and tables jvi.01327-25-s0001.pdf Figures S1 to S6; Tables S1 and S2.

ASM does not own the copyrights to Supplemental Material that may be linked to, or accessed through, an article. The authors have granted ASM a non-exclusive, world-wide license to publish the Supplemental Material files. Please contact the corresponding author directly for reuse.
