# Supplementary material for: Direct airway delivery of a humanized anti-H7N9 neutralizing antibody broadly protects against divergent H7 influenza viruses in the mouse model
Source: J Virol. 2025 Nov 24;99(12):e01327-25. doi: 10.1128/jvi.01327-25 (PMC12724141; doi:10.1128/jvi.01327-25)
Supplement: Supplemental figures and tables — Figures S1 to S6; Tables S1 and S2. [file jvi.01327-25-s0001.pdf]

A

Percent Identity (upper triangle); Divergence (lower triangle)

Divergence	1	2	3		
1		97.5	98.8	1	H7N9-GD15
2	2.5		97.3	2	H7N9-maSDL124
3	1.3	2.7		3	H7N9-SF003
	1	2	3		

B

Percent Identity (upper triangle); Divergence (lower triangle)

Divergence	1	2	3	4	5		
1		90.9	80.7	81.9	81.6	1	H7N2-fel2016
2	9.7		83.9	85.7	85.2	2	H7N3-Mex2012
3	22.4	18.1		94.1	93.4	3	H7N9-GD15
4	20.7	15.9	6.1		95.2	4	H7N4-JS2018
5	21.2	16.6	6.9	5.0		5	H7N2-DK2007
	1	2	3	4	5		

FIG S1 Identity of HA amino acid sequences among the H7 viruses used in this study. (A) Identity among three H7N9 viruses. (B) Identity among different H7 viruses. HA sequences were aligned using MegAlign in Lasergene software and pair distances among the sequences were shown.

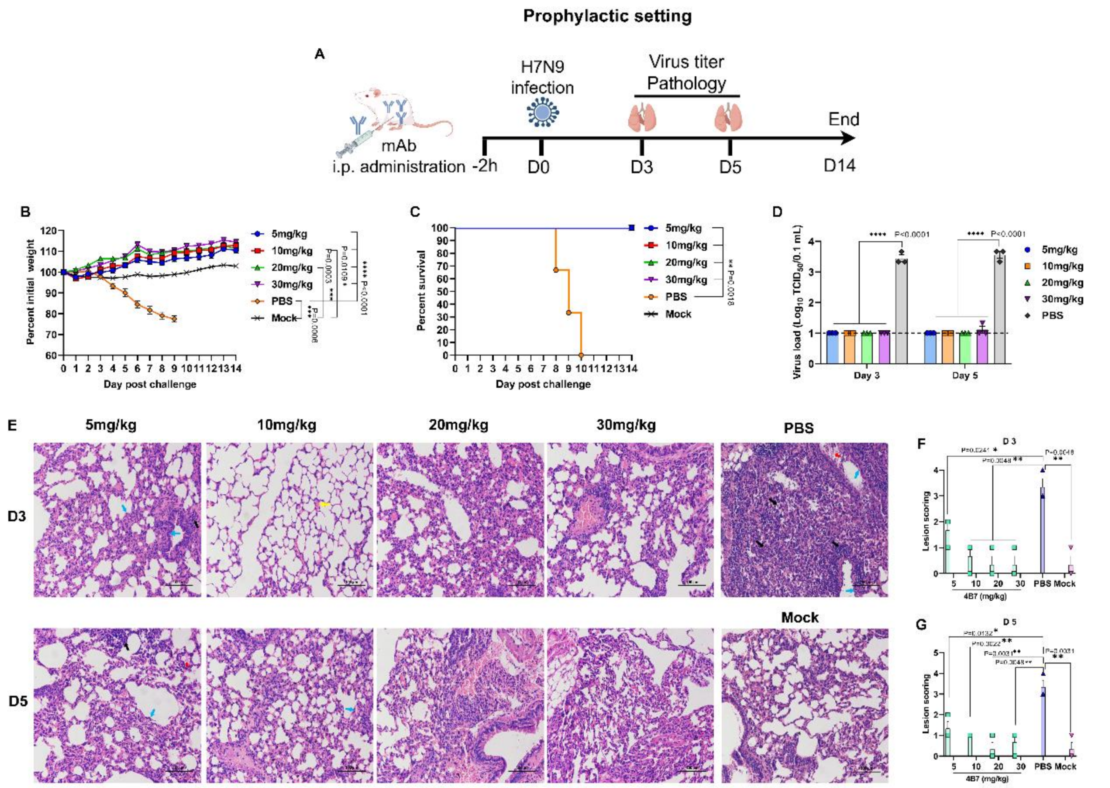

FIG S2 Prophylactic efficacy of the mAb 4B7 in mice via the intraperitoneal administration. (A) Study design. Groups of mice (n = 11) were administered with the mAb 4B7 at 30, 20, 10 and 5 mg/kg or PBS via the intraperitoneal route 2 h before infection with 10 MLD_{50} of the H7N9 maSDL124 virus. Mice (n = 5) were used as the mock control. Body weight (B) and survival (C) of the mice. (D) Virus loads in the lungs at day 3 and 5 post challenge. The lungs were collected (n = 3) and virus titers ($TCID_{50}$) were measured in MDCK cells. The dashed line indicates the detection limit of virus titration assay (10 $TCID_{50}$/0.1 mL). Values below the detection limit were shown as 1 $\log_{10}$ $TCID_{50}$/0.1 mL. (E) Lung pathology. The lungs tissue sections were prepared for H&E staining and histopathological examination. The representative photomicrographs of each group were shown. The black arrows indicate infiltration of lymphocytes and neutrophils; the red arrows indicate cell debris in the lumen of bronchus; the blue arrows stand for incrassation of alveolar wall; the yellow arrows stand for accumulation of erythrocytes. Scale bar, 100 µm. Lung lesions (F, G) were scored according to the following criteria: no or slight lesions, 0; mild lesions, 1; moderate lesions, 2; severe lesions, 3; very severe lesions, 4. Mean values ± standard error of the mean (SEM) of body weight (n = 5) and virus load or pathology (n = 3) per group were shown and analyzed using one-way ANOVA with Tukey's multiple comparison test. Survival between indicated two groups was analyzed using Log-rank (Mantel-Cox) test. Asterisks stand for significant differences and *P* values were shown.

Therapeutic setting

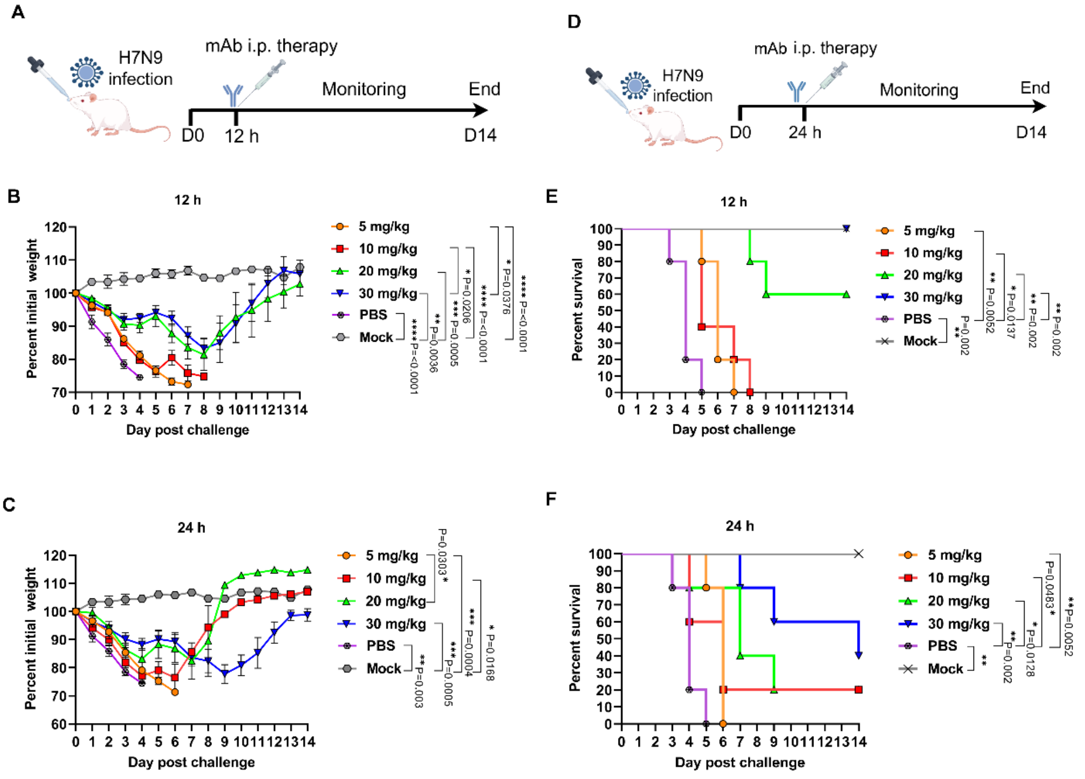

FIG S3 Therapeutic efficacy of the mAb 4B7 in mice via the intraperitoneal administration. (A, D) Study design. Groups of mice (n = 5) were infected with 10 MLD_{50} of the H7N9 maSDL124 and then treated with the antibody at 30, 20, 10 and 5 mg/kg or PBS via the intraperitoneal route at 12 h (A) or 24 h (D) post infection. Five mice were left un-treated and used as the mock control. Body weight (B, C) and survival (E, F) of the mice. Mean values ± standard error of the mean (SEM) of body weight (n = 5) per group were shown and analyzed using one-way ANOVA with Tukey's multiple comparison test. Survival between indicated two groups was analyzed using Log-rank (Mantel-Cox) test. Asterisks stand for significant differences and *P* values were shown.

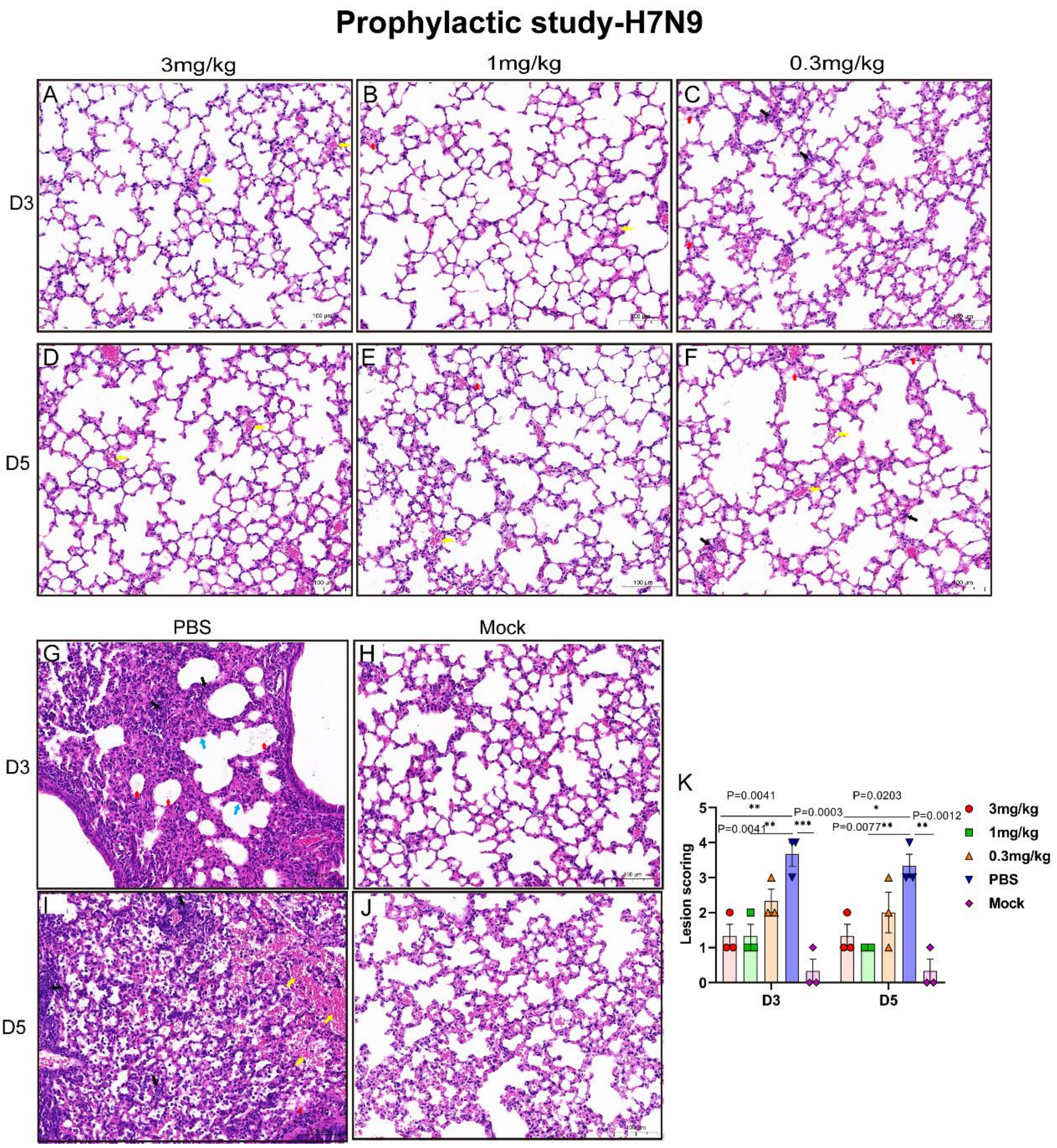

FIG S4 Lung pathology of the mice treated with chi4B7 before H7N9 virus infection. (A-J) Lung photomicrographs of the mice prophylactically administered with chi4B7 (3, 1 and 0.3 mg/kg) via the i.n. route before H7N9 infection. Lung tissues were collected from mice (n = 3) at day 3 and 5 post infection. The black arrows indicate infiltration of lymphocytes and neutrophils; the red arrows indicate cell debris in the lumen of bronchus; the blue arrows stand for incrassation of alveolar wall; the yellow arrows stand for accumulation of erythrocytes. Scale bar, 100 µm. (K) Lung lesions were scored according to the following criteria: no or slight lesions, 0; mild lesions, 1; moderate lesions, 2; severe lesions, 3; very severe lesions, 4. Mean scores ± standard error of the mean (SEM) of lesion scores (n = 3) per group were shown and analyzed using one-way ANOVA with Tukey's multiple comparison test. Asterisks stand for significant differences and *P* values were shown.

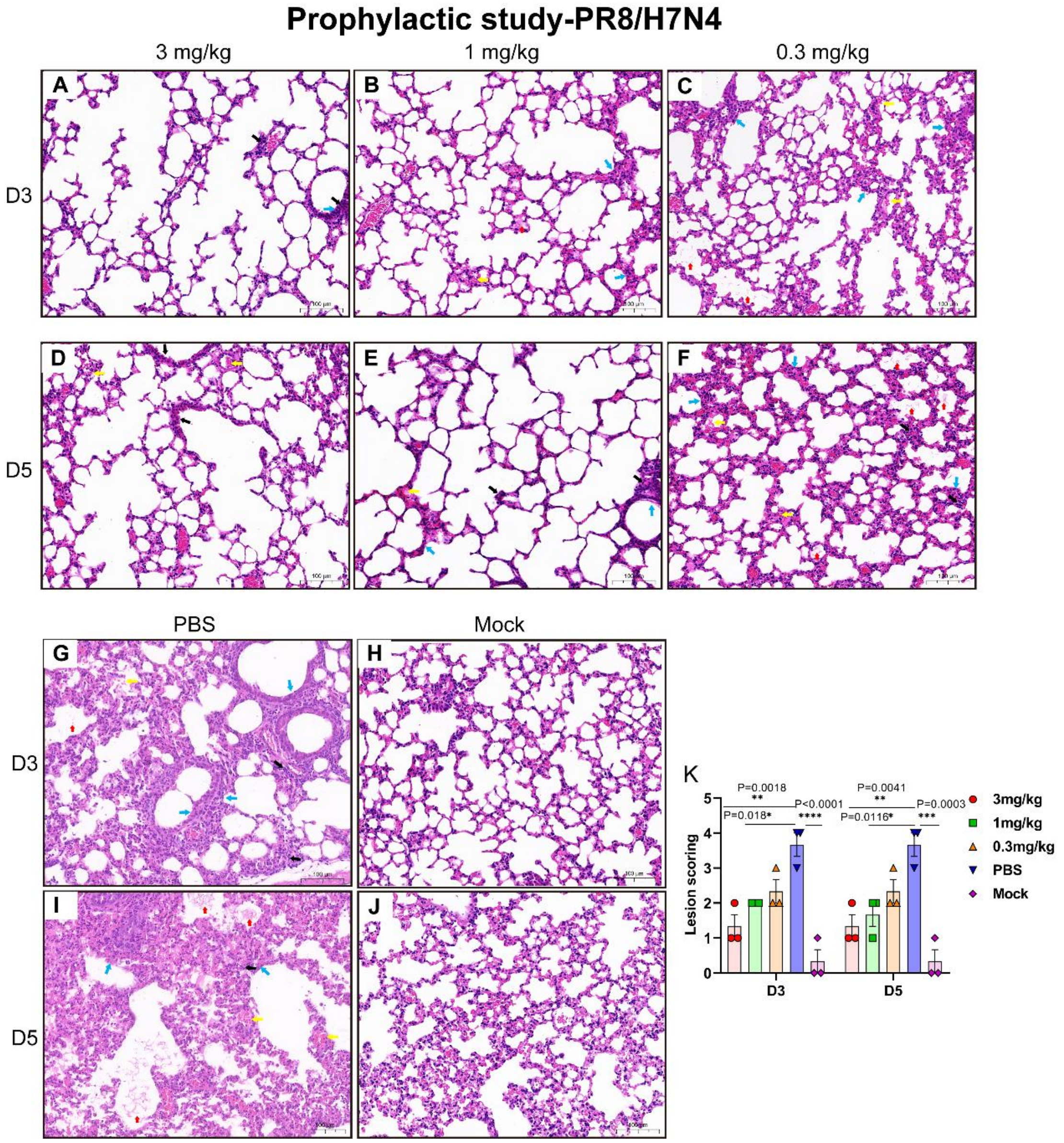

FIG S5 Lung pathology of the mice administered with chi4B7 before PR8/H7N4 virus infection. (A-J) Lung photomicrographs of the mice prophylactically administered with chi4B7 (3, 1 and 0.3 mg/kg) via the intranasal route before PR8/H7N4 infection. The lungs were collected from three mice at day 3 and 5 post infection. The black arrows indicate infiltration of lymphocytes and neutrophils; the red arrows indicate cell debris in the lumen of bronchus; the blue arrows stand for incrassation of alveolar wall; the yellow arrows stand for accumulation of erythrocytes. Scale bar, 100 μm. (K) Pathology was scored based on the following criteria: no or slight lesions, 0; mild lesions, 1; moderate lesions, 2; severe lesions, 3; very severe lesions, 4. Mean scores ± standard error of the mean (SEM) of lesion scores (n = 3) per group were shown and analyzed using one-way ANOVA with Tukey's multiple comparison test. Asterisks stand for significant differences and *P* values were shown.

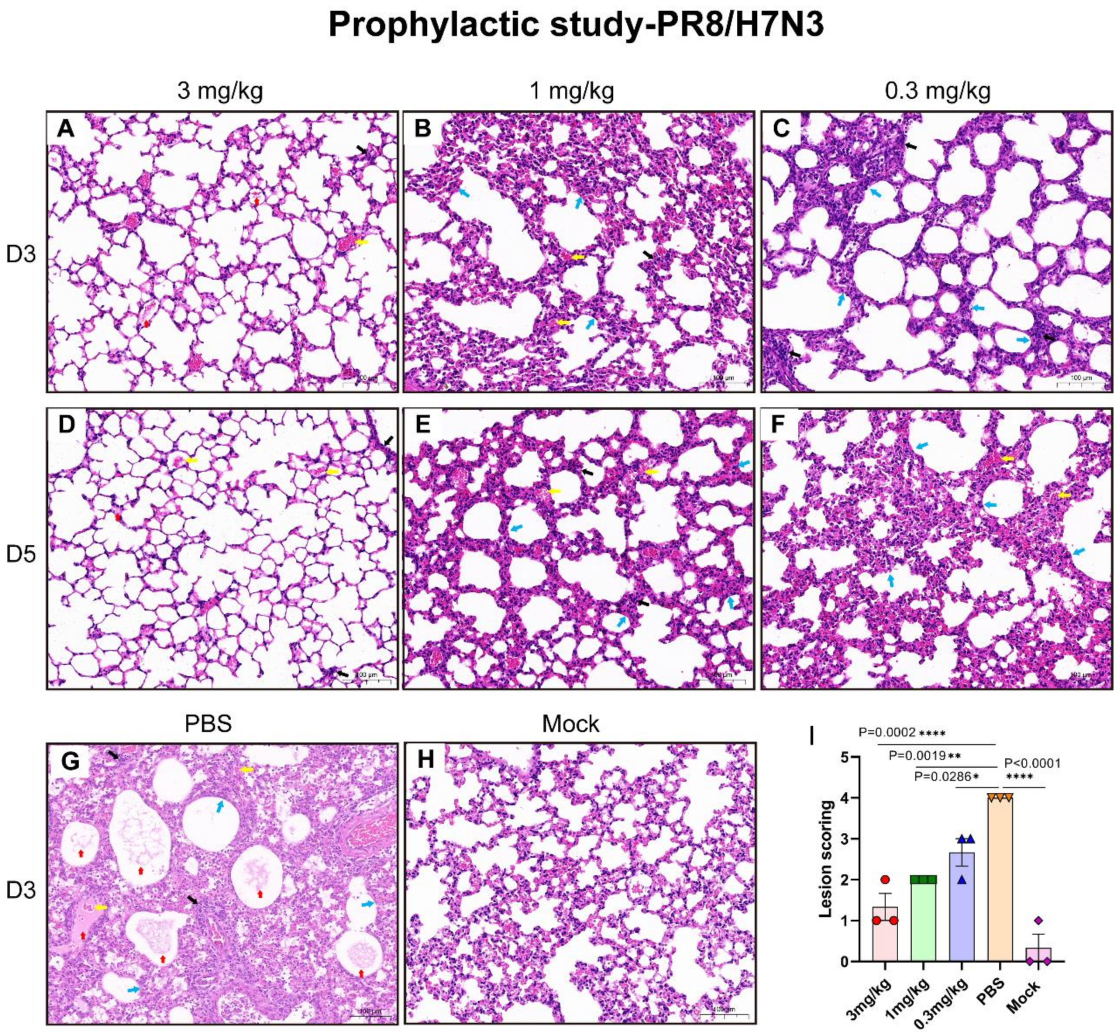

FIG S6 Lung pathology of the mice administered with chi4B7 before PR8/H7N3 virus infection. (A-H) Lung photomicrographs of the mice prophylactically administered with chi4B7 (3, 1 and 0.3 mg/kg) via the intranasal route before PR8/H7N3 infection. The lungs were collected from antibody-treated mice (n = 3) at day 3 post challenge (p.c.). Lung pathology of the PBS-treated mice (n = 3) at day 3 p.c. were shown because no mice survived beyond day 4 p.c. Therefore, pathology of the lungs collected at day 3 p.c. was shown. The black arrows indicate infiltration of lymphocytes and neutrophils; the red arrows indicate cell debris in the lumen of bronchus; the blue arrows stand for incrassation of alveolar wall; the yellow arrows stand for accumulation of erythrocytes. Scale bar, 100 μm. (I) Lung pathological lesions were scored based on the following criteria: no or slight lesions, 0; mild lesions, 1; moderate lesions, 2; severe lesions, 3; very severe lesions, 4. Mean scores ± standard error of the mean (SEM) of lesion scores (n = 3) per group were shown and analyzed using one-way ANOVA with Tukey's multiple comparison test. Asterisks stand for significant differences and *P* values were shown.

Table S1 Residues involved in the contacts between H7N9 HA and 4B7 scFv by PISA analysis

Residues in HA[a]	HA domain[b]	BSA(Å^2)[c]	Interacting scFv domain[d]
P82	VED	4.01	VH
Q83	VED	42.25	VH
D85	VED	40.6	VH+VL
Q86	VED	3.57	VH+VL
L88	VED	45.61	VL
E89	VED	103.29	VL
V104	VED	1.01	VH
D127		15.51	VL
K128		25.26	VL
E129		66.08	VL
P130		10.27	VL
M131		5.97	VH+VL
G132		42.23	VL
F133		14.32	VH
T134		38.77	VH
Y135		8.92	VH
N136		1.84	VH
R139	RBS	91.54	VH
T140	RBS	23.13	VH
N141	RBS	105.72	VH
G142	RBS	9.34	VH
V143	RBS	57.51	VH
R148	RBS	6.43	VH
R149		100.57	VH
S150		92.68	VH
G151		24.43	VH
S152	RBS	15.77	VH
S153		17.07	VH
F154		12.40	VH
Y155		1.11	VH
A156		22.99	VH
E157		1.47	VL
M158		8.96	VH
L159		27.37	VH
W160	RBS	0.29	VH
L162	RBS	14.39	VH
N164	RBS	1.72	VH
T165	RBS	47.36	VH
K175		1.18	VH
Y177		32.75	VL
K178		5.64	VL
T180		10.55	VL
R181		55.82	VL
A185		2.51	VL
D264		1.35	VH
R265		20.41	VL
A266		2.77	VL
F268		7.96	VL

[a]: amino acids were shown based on H7 numbering.

[b]: VED, vestigial esterase domain; RBS, receptor binding site.

[c]: buried surface area.

[d]: VH, variable heavy chain; VL, variable light chain.

Table S2 Hydrogen and salt bridge bonds between 4B7 scFv and H7N9 HA by PISA program

HA residues[a]	HA domain[b]	Chain[c]	Residues in scFv	Type[d]	Distance (Å)
K128		VL	S114(LCDR3)	H	3.68
E129		VL	S33(LCDR1)	H	3.44
P130		VL	N34(LCDR1)	H	3.41
M131		VH+VL	Y112(HCDR3)	H	2.44
			N34(LCDR1)	H	2.54
G132		VL	Y38(LCDR1)	H	3.83
F133		VH	F111(HCDR3)	H	2.88
			Y112(HCDR3)	H	2.34
T134		VH	F111(HCDR3)	H	3.60
R139	RBS	VH	D36(HCDR1)	H	2.74
				S	2.74
T140	RBS	VH	D57(HCDR2)	H	3.61
			S59(HCDR2)	H	3.13
			W38(HCDR1)	H	3.71
N141	RBS	VH	S63(HCDR2)	H	3.79
V143	RBS	VH	D62(HCDR2)	H	3.39
R149		VH	T65(HCDR2)	H	3.20
			Y67(HCDR2)	H	3.56
S150		VH	R72(HCDR2)	H	2.30
			Y67(HCDR2)	H	3.18
S152	RBS	VH	T65(HCDR2)	H	2.10
F154		VH	T65(HCDR2)	H	3.82
			Y64(HCDR2)	H	3.82
Y155		VH	Y64(HCDR2)	H	3.22
L159		VH	Y112(HCDR3)	H	2.12
T165	RBS	VH	S59(HCDR2)	H	3.15
K175		VH	F111(HCDR3)	H	3.48
Y177		VL	S32(LCDR1)	H	3.34

[a]: amino acids were shown based on H7 numbering.

[b]: VED, vestigial esterase domain; RBS, receptor binding site.

[c]: VH, variable heavy chain; VL, variable light chain.

[d]: H, hydrogen bond; S, salt bridge bond.

Table S3 Characterization of the mAb escape mutants

Virus	4B7 HI titer (log_2)	Residues in the HA protein	
		151	335
GD15	9	G	I
Escape mutants	5	E	V

Table S4 Characterization of the reassortant H7 viruses

Virus	Surface genes	Internal genes	HA titer (log_2)	$Log_{10}(EID_{50}/mL)$[a]	$Log_{10}(TCID_{50}/mL)$[b]	MLD_{50} (log_{10} $EID_{50}/50\mu L$)[c]
PR8/H7N4	H7+N4	PR8	8	8.0	2.5	4.38
PR8/H7N3	H7+N3	PR8	7	7.63	6.5	4.70
PR8/felH7N2	H7+N2	PR8	7	N.D.	3.5	N.D.
PR8/SF003	H7+N9	PR8	9	8.5	3.5	N.D.

[a]: EID_{50}, 50% embryo infectious dose; N.D., not determined.

[b]: $TCID_{50}$, 50% tissue culture infectious dose in MDCK cells.

[c]: MLD_{50}, 50% mouse lethal dose in log_{10} $EID_{50}/50\mu L$.
